# Heme Oxygenase-1, Oxidation, Inflammation, and Atherosclerosis

**DOI:** 10.3389/fphar.2012.00119

**Published:** 2012-07-19

**Authors:** Jesus A. Araujo, Min Zhang, Fen Yin

**Affiliations:** ^1^Division of Cardiology, Department of Medicine, David Geffen School of Medicine, University of CaliforniaLos Angeles, CA, USA

**Keywords:** heme oxygenase, bilirubin, carbon monoxide, iron, oxidative stress, inflammation, atherosclerosis

## Abstract

Atherosclerosis is an inflammatory process of the vascular wall characterized by the infiltration of lipids and inflammatory cells. Oxidative modifications of infiltrating low-density lipoproteins and induction of oxidative stress play a major role in lipid retention in the vascular wall, uptake by macrophages and generation of foam cells, a hallmark of this disorder. The vasculature has a plethora of protective resources against oxidation and inflammation, many of them regulated by the Nrf2 transcription factor. Heme oxygenase-1 (HO-1) is a Nrf2-regulated gene that plays a critical role in the prevention of vascular inflammation. It is the inducible isoform of HO, responsible for the oxidative cleavage of heme groups leading to the generation of biliverdin, carbon monoxide, and release of ferrous iron. HO-1 has important antioxidant, antiinflammatory, antiapoptotic, antiproliferative, and immunomodulatory effects in vascular cells, most of which play a significant role in the protection against atherogenesis. HO-1 may also be an important feature in macrophage differentiation and polarization to certain subtypes. The biological effects of HO-1 are largely attributable to its enzymatic activity, which can be conceived as a system with three arms of action, corresponding to its three enzymatic byproducts. HO-1 mediated vascular protection may be due to a combination of systemic and vascular local effects. It is usually expressed at low levels but can be highly upregulated in the presence of several proatherogenic stimuli. The HO-1 system is amenable for use in the development of new therapies, some of them currently under experimental and clinical trials. Interestingly, in contrast to the HO-1 antiatherogenic actions, the expression of its transcriptional regulator Nrf2 leads to proatherogenic effects instead. This suggests that a potential intervention on HO-1 or its byproducts may need to take into account any potential alteration in the status of Nrf2 activation. This article reviews the available evidence that supports the antiatherogenic role of HO-1 as well as the potential pathways and mechanisms mediating vascular protection.

## Heme Oxygenase-1 and Vascular Inflammation: Importance of Basal Levels

**Figure 1:**
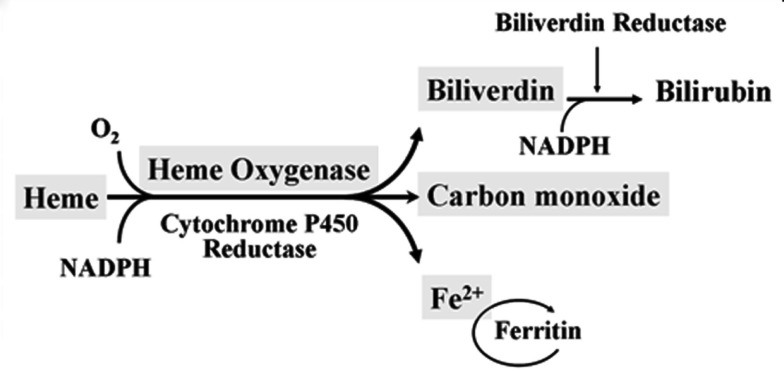
**Heme oxygenase enzymatic activity**. HO enzymatic leads to the generation of biliverdin, release of carbon monoxide (CO) and Fe^2+^. Biliverdin is transformed into bilirubin by the biliverdin reductase (BVR) enzyme. Fe^2+^ can be bound by the iron storage protein ferritin.

Heme oxygenase (HO) is a rate-limiting enzyme in the catabolism of heme. In association with cytochrome P450 reductase and in the presence of NADPH and three molecules of molecular oxygen (O_2_) per heme molecule, it catalyzes the oxidative cleavage of heme (Fe-protoporphyrin-IX) to render equimolar amounts of biliverdin, ferrous iron (Fe^2+^), and carbon monoxide (CO; Maines, 1997; Figure 1). Biliverdin can then be converted to bilirubin by the cytosolic enzyme biliverdin reductase (BVR). There are three HO isoforms (HO-1, HO-2, HO-3) that have been described (Siow et al., 1999), although HO-3 may be a pseudogene derived from HO-2 transcripts (Hayashi et al., 2004). While HO-2 is constitutively expressed, HO-1 is normally expressed at low levels in most tissues but it is highly inducible by a variety of stimuli. Indeed, HO-1 may be among the most critical cytoprotective mechanisms that are activated during times of cellular stress such as inflammation, ischemia, hypoxia, hyperoxia, hyperthermia, or radiation (Choi and Alam, 1996). It is thought to play a key role in maintaining antioxidant/oxidant homeostasis and in the prevention against vascular injury (Abraham and Kappas, 2008).

**Figure 2:**
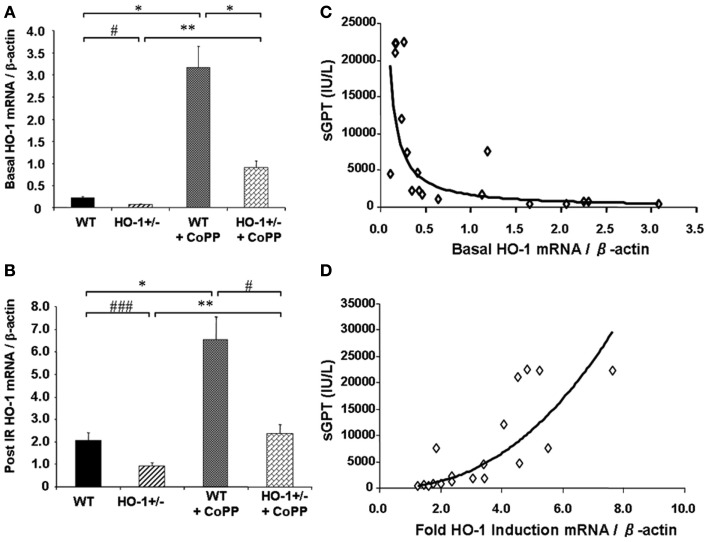
**Basal HO-1 protects against oxidation and inflammation**. HO-1^+/−^ and HO^+/+^ (WT) mice were subjected to partial liver warm ischemia for 90 min followed by 6 h of reperfusion. Two groups of mice were treated with CoPP. **(A)** Basal HO-1 mRNA levels, **(B)** Post-ischemia reperfusion (IR) HO-1 mRNA levels, **(C)** Basal HO-1 levels correlated negatively with post-IR serum GPT levels (*R*^2^ = 0.69, *p* < 0.0001) **(D)** HO-1 fold induction had a positive correlation with sGPT levels instead (*R*^2^ = 0.75, *p* = 0.0001). Taken from Tsuchihashi et al. (2006). Copyright 2006. The American Association of Immunologists, Inc.

Cumulative evidence has shown that expression of HO-1 in the vasculature exerts protective effects against vascular inflammatory processes, thanks to its antioxidant, antiinflammatory, antiapoptotic, and possibly immunomodulatory properties. Thus, HO-1 antioxidant protection is particularly evident against the vascular inflammation occurring in models of antigen-independent ischemia reperfusion injury (IRI), where the reintroduction of oxygen after a period of ischemia and generation of reactive oxygen species (ROS) constitutes the central pathogenic event. Pharmacological competitive inhibitors of HO activity such as the heme analogs SnPPIX or ZnPPIX (Kato et al., 2001) or decreased HO-1 expression in genetically engineered HO-1^+/−^ mice lead to significant worsening of IRI outcomes (Tsuchihashi et al., 2006). On the other hand, HO-1 overexpression by either pharmacological means or via genetic engineering has been reported to exert potent cytoprotective effects in hepatic IRI transplant models, with profoundly diminished proinflammatory and apoptotic responses (Amersi et al., 1999; Kato et al., 2001; Coito et al., 2002). Of importance, basal HO-1 levels, rather than the degree of HO-1 upregulation, appear to be crucial in the antioxidant cytoprotection as indicated by data obtained from HO-1^+/−^ and HO-1^+/+^ mice subjected to partial liver warm ischemia for 90 min followed by 6 h of reperfusion (Tsuchihashi et al., 2006; Figure 2). Liver IRI injury is characterized by hepatocellular damage consisting in neutrophil infiltration, sinusoidal congestion, hepatocyte apoptosis/necrosis, and ballooning degeneration as well as impaired hepatocyte function that can be determined by elevation of serum transaminases such as the serum glutamic-oxaloacetic transaminase (sGOT) and the glutamic-pyruvic transaminase (sGPT). Treatment with CoPP (Cobalt Protoporhyrin), a synthetic analog of heme that induces HO-1 without competing for its catalytic site, led to upregulation in both basal (Figure 2A) and post-IRI HO-1 mRNA levels (Figure 2B) that led to decreased IRI overall, evidenced by decreased post-IRI serum transaminases. Interestingly, both basal (Figure 2C) and post-IRI HO-1 mRNA levels (Tsuchihashi et al., 2006) correlated negatively with sGPT levels but the degree of correlation was bigger with the basal (*R*^2^ = 0.87, *p* = 0.0002) than with the post-IRI HO-1 levels (*R*^2^ = 0.55, *p* = 0.0002). In addition, sGPT levels correlated positively with the degree of HO-1 fold induction (Figure 2D), suggesting that the basal HO-1 levels (Figure 2A) may be more important than the degree of upregulation. However, this could also be due to the HO-1 behavior as a stress-responsive gene since it can be rapidly and highly upregulated by a large variety of acute stressors. Therefore, the degree of HO-1 fold induction might reflect the level of stress induced in the liver by the ischemia reperfusion rather than an ability to protect against the injury.

Heme oxygenase-1 antiinflammatory properties are clearly evident in models of organ transplantation, not only as a consequence of the protection against IRI but also by protecting against antigen-dependent immune responses. Thus, pharmacological inhibition (Hancock et al., 1998) or decreased HO-1 expression in mouse cardiac allografts lead to accelerated acute rejection, in parallel with similar results in mouse to rat xenografts (Soares et al., 1998). In addition, HO-1 overexpression in the cardiac allograft results in prolongation of the graft survival, which is much more dramatically improved when HO-1 is overexpressed in the recipient host rather than the donor organ (Araujo et al., 2003). It is possible then that HO-1 antiinflammatory properties involve specific immunomodulatory actions that could affect antigen presentation and/or shifting of the type of cellular immune response (Araujo et al., 2003; Chauveau et al., 2005). Interestingly, in a mouse model of heterotopic cardiac allograft transplantation, where C57BL/6J hearts were transplanted into BALB/cByJ recipients, hearts from HO-1^±^ mice, with HO-1 mRNA levels ~60% of wild-type (WT) controls displayed shorter survival, despite a much larger degree of HO-1 upregulation after the transplant (unpublished), suggesting that basal HO-1 could be key in antiinflammatory protection. However, the different degree of HO-1 fold induction in HO-1^±^ mice could also reflect here, as it does in the case of the IRI, a greater level of stress induced in the transplanted hearts by various inflammatory stressors rather than a different ability to protect against the rejection response.

**Figure 3:**
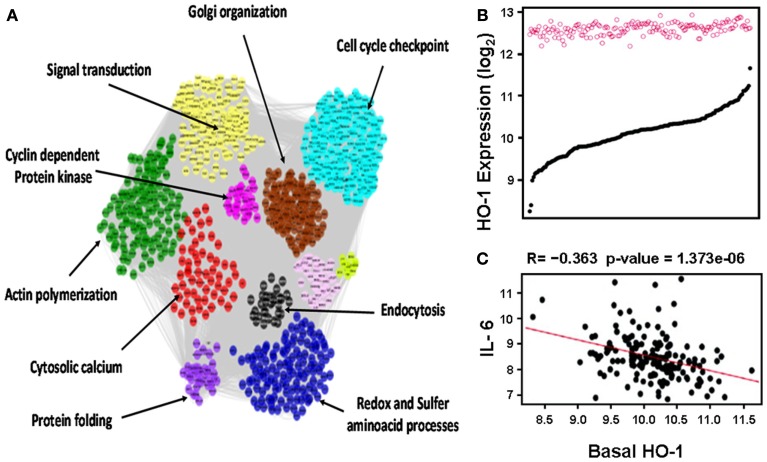
**Heme oxygenase-1 is a critical gene in the activation of endothelial cells by oxPAPC**. **(A)** Visualization of the whole network consisting of 2000 transcripts, organized into 11 modules, shown by distinct colors. Network was constructed based on genomic expression data of HACEs from 149 donors, treated with oxPAPC. Examples of module enrichment in specific pathways are shown. HO-1 is a “hub” of gene-gene interactions in the blue module, enriched in redox and sulfur aminoacid processes. **(B)** HO-1 mRNA expression. Basal levels varied approximately ninefold at baseline (black curve) but only approximately twofold after treatment with oxPAPC (red circles). **(C)** Basal HO-1 mRNA levels correlated negatively with IL-6 mRNA expression. Taken from Romanoski et al. (2011).

This notion was further confirmed in human aortic endothelial cells (HAECs) from 149 individual donors treated with oxidized 1-palmitoyl-2-arachidonyl-*sn*-glycero-3-phosphorylcholine (oxPAPC) as mentioned in more details below (Romanoski et al., 2011; Figure 3). In brief, baseline HO-1 mRNA levels, as judged by HAECs treated with media alone, exhibited a significant variation among different donors whereas treatment with oxPAPC resulted in significant upregulation of HO-1 levels that unlike baseline levels vary little in between donor cells (Figure 3B). oxPAPC treatment also led to the upregulation of several proinflammatory cytokines such as Interleukin (IL)-6. There was a negative correlation between basal HO-1 and levels of proinflammatory cytokines (Figure 3C). In addition, basal HO-1 levels were highly correlated with individual responsiveness to oxPAPC treatment at thresholds above 2-fold and 7.5-fold (*R* = −0.57) when responsiveness was defined as the number of genes that were regulated above a given threshold per individual. This suggests that basal HO-1 levels are important in the protection against inflammation and in mitigating multiple cellular responses to oxPAPC (Romanoski et al., 2011).

The greater importance of basal HO-1 levels as compared to the level of upregulation has important implications, especially since these observations have been made in human primary cells that did not undergo any genetic manipulation. First, if basal HO-1 expression is an important protective factor against vascular inflammation, why are the higher levels following induction unable to abrogate the inflammatory process? Second, how functional is the induced HO-1 protein? Third, what would be the ideal timing for potential therapeutic interventions and how should HO-1 responses be assessed, by changes in the level of mRNA, protein, functional activity, or levels of its enzymatic byproducts? While it appears to be clear that HO-1 functional activity is important for its vascular protection as will be discussed in Section “HO System, Enzymatic Byproducts and Protection Against Atherosclerosis,” there is a possibility that various stimuli or microenvironments could lead to changes in the specific enzymatic activity, which is the HO-1 enzymatic activity/HO-1 protein. Thus, Romanoski et al. (2011) showed that while oxPAPC treatment led to increased HO-1 mRNA, protein, and enzymatic activity in HAECs, the HO activity increased in a lesser degree than the HO-1 protein, suggesting a lower HO specific activity after treatment with oxPAPC as compared with basal levels. This is consistent with another study that showed a disparity between HO-1 protein and HO activity in a rat model of metabolic syndrome where HO-1 protein was likely modified by nitrosylation resulting in decreased functionality (Kruger et al., 2006). While our current understanding of the biology of HO-1 may not be sufficient to answer all these questions at the present time, the following sections attempt to provide information that we hope will help to address them.

## Role of HO-1 and Other Antioxidant Genes in Atherosclerosis

Atherosclerosis is an inflammatory process of the vascular wall characterized by the accumulation of lipids and fibrous elements in large and medium-sized elastic and muscular arteries (Lusis, 2000). Infiltrating lipids come from circulating low-density lipoprotein (LDL) particles that are retained in the vascular wall. The retention of LDL is favored by its oxidative modification which leads to activation of endothelial cells, monocyte recruitment with internalization into the vasculature, differentiation into macrophages and generation of foam cells by increased lipid uptake. Vascular infiltration of lipids and inflammatory cells further enhances oxidative stress and a vicious cycle of inflammation (Araujo et al., 2002). While the pathogenic role of vascular oxidative stress has been challenged by the argument that oxidative modifications present in the plaques could be consequential rather than causal to lesion formation (Stocker and Keaney, 2004), it does appear that the interplay between prooxidant and antioxidant factors in the vasculature may determine the degree of ROS generation in a way that can affect lesion formation. Thus, established risk factors such as diabetes and cigarette smoking or novel risk factors such as exposure to air pollution enhance ROS generation in the vasculature and promote atherogenesis (Araujo, 2011). Part of these actions may be mediated by activation of NADPH oxidase, which has been shown to be an important source of vascular ROS and deficiency of its p47phox subunit results in decreased atherosclerotic lesion formation in ApoE null mice (Barry-Lane et al., 2001). On the other hand, organisms have numerous antioxidant resources that may protect against increased ROS formation in the vasculature. Some of them are in the circulating blood such as albumin, bilirubin, or plasma high-density lipoproteins (HDL) that can exert antioxidant protection. Others are within vascular cells and include a large number of antioxidant genes and phase-2 detoxifying enzymes regulated by the transcription factor Nrf2 (NFE2 related factor 2). HO-1 is among those Nrf2-target genes, which is significantly expressed in all main cell types present in mouse and human atherosclerotic lesions, such as endothelial cells (EC), macrophages, and smooth muscle cells (SMCs; Wang et al., 1998; Ishikawa et al., 2001b).

### Heme oxygenase-1 and other antioxidant genes protect against atherosclerosis

The importance of HO-1 expression in the protection against human atherosclerotic lesions has been emphasized by various genetic population studies, which have shown that a GT length polymorphism in the promoter region of the human HO-1 gene is related to susceptibility for atherosclerosis. Thus, a shorter number of repeats has been associated with decreased susceptibility to coronary artery disease (CAD) in diabetic Japanese (Kaneda et al., 2002) and Chinese (Chen et al., 2002) populations, abdominal aortic aneurysms (Schillinger et al., 2002) and post-angioplasty restenosis, in both coronary (Chen et al., 2004) and peripheral arteries (Exner et al., 2001) in comparison with a longer number of repeats. Short GT variants (S) are likely to result in increased HO-1 expression in comparison with long variants (L) as judged by reporter transfection assays (Yamada et al., 2000), even though the cutoff to differentiate between S and L variants have been different among various studies. This was confirmed in human umbilical vein endothelial cells (HUVECs). Cells carrying the S allele exhibited higher levels of HO-1 mRNA, protein, and HO activity than cells carrying the L allele (Taha et al., 2010). While HO-1 deficiency is very rare in humans, additional confirmation of the importance of HO-1 against vascular inflammation derives from the first autopsy report of a HO-1 deficient 6-year-old boy who exhibited hyperlipidemia (Yachie et al., 1999), foamy macrophages in the liver as well as fatty streaks and fibrous plaques in the aorta (Kawashima et al., 2002). In addition, HO-1 deficiency led to very high concentrations of circulating heme and damage to vascular endothelium that was probably mediated through the generation of oxidized forms of LDL (Jeney et al., 2002) which could have contributed to atherosclerotic lesion formation. It is unclear however whether these changes can be solely attributed to HO-1 deficiency since this boy had undergone a long-term steroid treatment for a presumptive diagnosis of juvenile rheumatoid arthritis that was not confirmed upon pathological examination (Kawashima et al., 2002).

We and others have shown that HO-1 is an antiatherogenic gene in animal models (Ishikawa et al., 2001a,b; Juan et al., 2001; Yet et al., 2003; Orozco et al., 2007) as summarized in Table 1. Systemic lack of HO-1 has been reported to promote atherosclerosis in ApoE null mice (Yet et al., 2003) as well as aortitis in chow-fed old C57BL/6 mice (Ishikawa et al., 2012), while its overexpression by adenoviral means results in decreased atherosclerotic lesions (Juan et al., 2001). Likewise, pharmacological manipulation of the gene results in similar effects. Thus, administration of hemin results in significant HO-1 upregulation and decreased atherogenesis in LDLR^−/−^ mice (Ishikawa et al., 2001b), ApoE^−/−^ mice (Cheng et al., 2009), and rabbits fed a high fat diet (Li et al., 2011; Liu et al., 2012). Since increased expression of HO-1 carries the danger of increased release of ferrous iron (Fe^2+^) and exacerbation of iron-mediated ROS formation, Ishikawa et al. (2001b) administered iron-chelating deferoxamine to LDLR^−/−^ mice that were treated with hemin to avoid this potential problem. However, subsequent studies in ApoE^−/−^ mice and rabbits showed that this was not necessary (Cheng et al., 2009; Li et al., 2011; Liu et al., 2012). This is likely due to the fact that HO-1 upregulation colocalizes with increased expression of ferritin in atherosclerotic lesions, which may ensure safe disposal of the released Fe^2+^.

**Table 1:** **Animal studies supporting HO-1 antiatherogenic role**.

Study/References	Experimental model	HO-1 modulation	Findings
Ishikawa et al. (2001a)	Watanabe rabbits on a chow diet	SnPPIX (−)	I.P. SnPPIX 5×/week for 5 weeks inhibited aortic HO activity, increased plasma, aortic, and liver lipoperoxides and enhanced aortic atherosclerotic plaques (en face) by 155% as compared with controls.
Ishikawa et al. (2001b)	LDL-R^−/−^ mice fed a chow diet or HFD	Hemin (+) SnPPIX (−)	I.P. hemin or hemin + deferoxamine 4×/week for 6 weeks induced aortic HO-1 and decreased aortic root lesions. I.P. SnPPIX decreased aortic HO activity and enhanced lesions as compared with controls.
Juan et al. (2001)	ApoE^−/−^ mice fed a chow diet	I.V. Adv HO-1 vs. L.V. Adv HO-1	I.V. Adv HO-1 for 1 week increased liver and aortic HO-1 and enhanced aortic root lesions while L.V. Adv HO-1 failed to increase aortic HO-1 and did not affect lesion formation as compared with controls. Similar findings in 14-week and 20-week old mice.
Yet et al. (2003)	HO-1^−/−^ApoE^−/−^ vs. HO-1^+/+^ ApoE^−/−^ mice on a HFD for 8 weeks	Genetic deletion of HO-1	12-week-old HO-1^−/−^ApoE^−/−^ mice, fed 8 weeks on HFD developed increased atherosclerotic lesions in the right brachiocephalic arteries by ~260% as compared with HO-1^+/+^, ApoE^−/−^ controls.
Orozco et al. (2007)	Bone marrow transplantation of HO-1^−/−^ vs. HO-1^+/+^ into LDL-R^−/−^ mice fed a HFD	Genetic deletion of HO-1 in bone marrow-derived cells	Sublethally irradiated LDL-R^−/−^ mice reconstituted with HO-1^−/−^ bone marrow developed aortic root plaques with greater macrophage content than control mice reconstituted with HO-1^+/+^ bone marrow. HO-1 expression decreased generation of foam cells in macrophages.
Cheng et al. (2009)	ApoE^−/−^ mice fed a HFD diet, with a cast around right carotid artery for 9 weeks	CoPPIX (+) ZnPPIX (−)	I.P. CoPPIX for 3 weeks increased relative cap thickness and decreased necrotic core/intima ratio while ZnPPIX resulted in the opposite despite no effects on plaque burden, suggesting that HO-1 promotes plaque stability.
Li et al. (2011)	New Zealand White Rabbits fed a HFD, subjected to balloon-induced aortic injury	hemin (+) SnPPIX (−)	I.P. hemin every other day for 12 weeks decreased intimal area positive for macrophages, lipids by 35 and 43% respectively, but increased intimal SMCs and collagen by 100 and 42% respectively. SnPPIX resulted in opposite results. Hemin decreased apoptosis and MMP-9 expression while SnPPIX resulted in the opposite.
Liu et al. (2012)	New Zealand White Rabbits, fed a chow diet and HFD	hemin (+) ZnPPIX (−)	I.P. ZnPPIX everyday for 12 weeks increased plaque area by 19% while hemin led to a threefold decrease in plaque area vs. controls fed a HFD. Hemin increased CO generation and decreased eNOS activity and NO tissue levels.

*HFD, high fat diet, I.V., intravenous, L.V., left ventricular, I.P., intraperitoneal, Adv, adenoviral*.

On the other hand, inhibition of HO enzymatic activity by SnPPIX leads to enhanced atherosclerotic lesions in LDL-R^−/−^ mice (Ishikawa et al., 2001b), Watanabe hyperlipidemic rabbits (Ishikawa et al., 2001a) and rabbits fed a high fat diet (Li et al., 2011; Liu et al., 2012; Table 1). In addition, inhibition of HO enzymatic activity by ZnPPIX augmented plaque vulnerability in ApoE null mice by decreasing the relative cap thickness and intimal surface area of SMC, at the same time of increasing the lipid content and the degree of core necrosis (Cheng et al., 2009). It is clear then that modulation of HO-1 expression significantly alters atherogenesis in various animal models. The different degrees how atherogenesis was affected in all these studies may have to do with differences in the experimental designs and the inherent characteristics of the animal models employed (Table 1).

As ROS generation and tissue oxidative stress have been implicated in all stages of atherosclerosis, it appears that HO-1 expression is protective against the development of both early and advanced atherosclerotic plaques. HO-1 antioxidant and antiinflammatory properties may be crucial against the development of early plaques (Ishikawa et al., 2001b, 2012; Orozco et al., 2007), while its antiapoptotic activities can be important in lesion progression and/or plaque vulnerability for rupture (Cheng et al., 2009; Li et al., 2011). Interestingly, HO-1 expression could inhibit maturation of dendritic cells (Chauveau et al., 2005) and may modulate antigen presentation and participate in macrophage differentiation/polarization, phenomena that could be particularly important in the development of early stage lesions. On the other hand, as intraplaque hemorrhage and accumulation of heme/iron are an important component in lesion progression, HO-1 expression could be important to ensure catabolism of the heme groups and recycling of iron, which could affect the development of advanced plaques as well.

Heme oxygenase-1 antiatherogenic properties parallel its vascular protective effects in other models of vascular inflammation such as post-angioplasty restenosis (Duckers et al., 2001; Tulis et al., 2001), allograft rejection (Araujo et al., 2003), and ischemia reperfusion (Tsuchihashi et al., 2006). Like HO-1, other Nrf2-regulated antioxidant genes such as glutamate-cysteine ligase, modifier subunit (Gclm), glutamate-cysteine ligase, catalytic subunit (Gclc), peroxiredoxin 2, glutathione peroxidase, have been shown to protect against atherosclerosis. Thus, while deficiency of Gclm accelerates advanced atherosclerosis in ApoE null mice, overexpression of Gclc results in the opposite (Callegari et al., 2011). Systemic deficiency of peroxiredoxin or in bone marrow-derived cells enhances atherosclerotic lesions in ApoE null mice (Park et al., 2011). Similarly, deficiency of glutathione peroxidase exacerbates diabetes mellitus-associated atherogenesis in ApoE null mice (Lewis et al., 2007). On the other hand, adenoviral administration of endothelial cell Superoxide dismutase (EC-SOD; Laukkanen et al., 2002; Brasen et al., 2007), CuZnSOD (Durand et al., 2005), and catalase (Durand et al., 2005) inhibit post-angioplasty restenosis instead. Overall, HO-1 and several antioxidant genes, all regulated by Nrf2, exert protection against atherosclerosis.

### Nrf2 transcription factor paradoxically promote atherosclerosis

The substantial evidence in support of the antiatherogenic role of several Nrf2-regulated antioxidant genes led to the hypothesis that this transcription factor should play an inhibitory role of atherogenesis as well. Surprisingly, we and others have reported three separate studies showing that Nrf2 deficiency in gene-targeted mice leads to decreased atherosclerotic lesions in ApoE null mice (Sussan et al., 2008; Barajas et al., 2011; Freigang et al., 2011), suggesting that its expression may play a proatherogenic role despite its antioxidant actions. Nrf2^−/−^ApoE^−/−^ mice developed reduced aortic atherosclerosis as compared to Nrf2^+/−^ApoE^−/−^ (Barajas et al., 2011; Freigang et al., 2011) or Nrf2^+/+^ApoE^−/−^ controls (Sussan et al., 2008; Barajas et al., 2011). The type of diet appears to be an important modulating factor as these effects were only seen in males when mice were fed a chow diet (Barajas et al., 2011).

**Figure 4:**
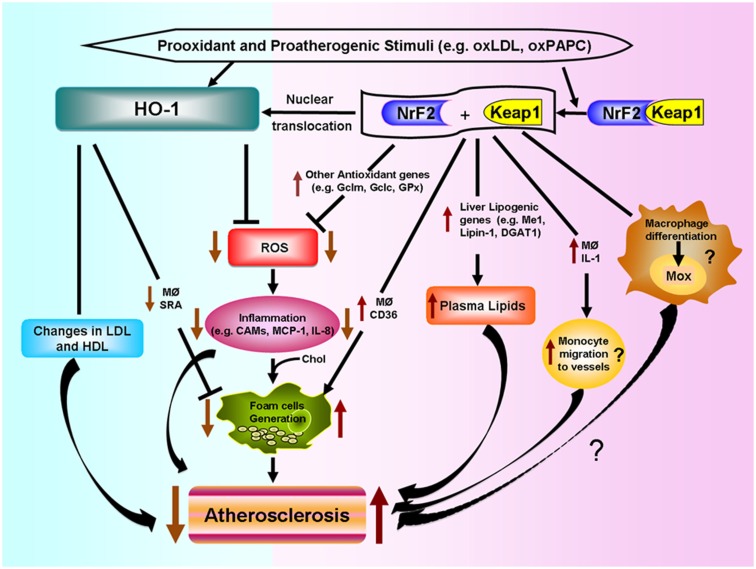
**Differential effects of HO-1 and NRF2 in atherosclerosis**. Prooxidant and proatherogenic stimuli such as oxLDL and oxPAPC can induce HO-1 expression in vascular cells via Nrf2 activation or other pathways. HO-1 expression leads to decreased ROS generation, decreased inflammatory events such as lower expression of cell adhesion molecules (CAMs) and decreased secretion of inflammatory factors such as monocyte chemotactic protein (MCP)-1 and IL-8. HO-1 also leads to decreased foam cell formation and changes in LDL and HDL lipoproteins, all of which lead to decreased atherogenesis. Nrf2 is bound by chaperone Keap1 in the cytosol. Electrophilic agents lead to dissociation of the Nrf2-Keap1 complex, with release of Nrf2 and nuclear translocation that leads to the induction of HO-1 and several other antioxidant genes that result in decreased ROS formation. However, Nrf2 expression promotes atherosclerotic lesion development. Possible mechanisms include: (1) increased macrophage lipid uptake and foam cell formation, (2) increased lipogenesis and greater levels of non-HDL plasma cholesterol, (3) greater macrophage secretion of IL-1 and monocyte migration to the vessels, and (4) possible macrophage differentiation into a proatherosclerotic phenotype.

Various putative mechanisms, as shown in Figure 4, could mediate Nrf2 proatherogenic effects as recently discussed in an editorial article (Araujo, 2012): (1) Nrf2 expression leads to increased levels of plasma non-HDL cholesterol in Chow-fed ApoE null mice, likely via enhancing liver lipogenesis (Huang et al., 2010; Barajas et al., 2011), (2) Nrf2 promotes foam cell formation, partially due to upregulation of the CD36 scavenger receptor (Ishii et al., 2004; Barajas et al., 2011), (3) Nrf2 enhances increased expression of IL-1α in macrophages which may promote greater monocyte migration to the lesions (Burns and Furie, 1998; Freigang et al., 2011), and (4) Nrf2 may regulate the differentiation of macrophages to subtypes different to the classical M1 and M2, named Mox (Kadl et al., 2010) and possibly Mhem (Boyle et al., 2011, 2012), with functions in atherosclerotic lesion formation still to be determined.

The opposite effects of HO-1 and Nrf2 in atherogenesis shown in Figure 4, underline the high degree of complexity of pathways involved in the response to prooxidative and proatherogenic stimuli. In addition, it appears that the level of regulation or intervention upon these responses is quite important as downstream interventions such as deletion of antioxidant genes like HO-1 result in exacerbation of atherosclerosis but a more upstream level of intervention, such as deletion of Nrf2 transcription factor, results in the opposite. It is therefore quite important to understand how HO-1, as well as other antioxidant genes, exert their antiatherogenic effects.

## Heme Oxygenase-1 Antiatherogenic Effects: Systemic vs. Vascular Mechanisms

Heme oxygenase-1 is ubiquitously expressed and highly upregulated in all the main cell types present in human (Wang et al., 1998) and murine atherosclerotic lesions (Ishikawa et al., 2001b), including endothelial cells, macrophages, and SMCs, but it is very low in neighboring unaffected vascular tissue. Such upregulation is particularly noticeable in macrophages, foam cells, and endothelial cells (Ishikawa et al., 2001b). The overall protective effects of HO-1 have been well documented and the subject of recent reviews (Morita, 2005; Abraham and Kappas, 2008; Soares and Bach, 2009; Durante, 2011) but the importance of the tissue and cellular localization as well as precise mechanisms responsible for these effects remain unclear. Several studies have addressed the role of HO-1 in atherogenesis as mentioned in the previous section (Table 1). For the most part, these studies have used systemic modulation of either HO-1 expression and/or HO activity, which occurs in both vascular and non-vascular cells, with subsequent alteration of oxidant and inflammatory parameters in the circulating blood. It is possible that HO-1 antiatherogenic actions are due to a combination of systemic and local vascular mechanisms.

### Systemic effects

Systemic modulation of HO-1 expression levels or activity can lead to changes in the systemic levels of its enzymatic byproducts that could mediate some of the effects in the vasculature. Thus, systemic transgenic mice that overexpressed HO-1 by only ~30–50% in the liver over WT controls, exhibited a modest increase in blood unconjugated bilirubin (Araujo et al., 2003). Likewise, I.P. treatment of rabbits with hemin for 12 weeks led to increased HbCo levels while I.P. treatment with inhibitor SnPPIX for similar time led to the opposite (Li et al., 2011). In humans, small variations in bilirubin levels translate into clinical effects.

Changes in blood or vascular concentrations of HO byproducts could lead to alterations in plasma lipoproteins that may affect susceptibility to atherogenesis. Indeed, HO-1 null mice exhibit increased levels of plasma lipid peroxides (Ishikawa et al., 2012) that are parallel to their increased levels of lipid peroxides and protein carbonyls in the liver and kidneys (Poss and Tonegawa, 1997). Likewise, inhibition of HO activity by ZnPPIX leads to increased serum levels of oxLDL (Liu et al., 2012). It is possible that HO-1 deficiency or inhibition of HO enzymatic activity could lead to increased levels of circulating heme which may mediate lipid peroxidation in plasma lipoproteins, resulting in oxidized LDL (Balla et al., 1991) and lipid oxidation in atherosclerotic plaques (Nagy et al., 2010). Indeed, the 6-year-old boy, deficient in HO-1, was reported to have chronic intravascular hemolysis (Yachie et al., 1999) resulting in very high concentrations of circulating heme and a high proportion of methemoglobin (Jeney et al., 2002). The latter is a form of the oxygen-carrying hemoglobin that can not bind oxygen and releases iron in a greater degree than the normal oxy-hemoglobin with the subsequent potential of promoting oxidative injury and endothelial cell toxicity (Jeney et al., 2002). Therefore, HO-1 mediated disposal of heme groups may help to decrease systemic lipid peroxidation, generation of oxidized LDL and cellular injury (Figure 4).

Heme oxygenase-1 systemic effects also appear to involve HDL lipoproteins as HO-1 null mice exhibit decreased levels of paraoxonase activity and altered ratio of apoAI to apoAII proteins, which are likely to alter HDL function (Ishikawa et al., 2012). Therefore, HO-1 expression may protect against LDL oxidation or decrease its susceptibility to oxidation as it alters HDL protective qualities (Figure 4). In addition, HO-1 expression may also affect inflammatory mediators at a systemic level, as HO-1 null mice have been reported to exhibit greater plasma levels of monocyte chemotactic protein (MCP)-1 protein and increased levels of MCP-1 mRNA in circulating leukocytes as compared with wild-type mice (Pittock et al., 2005).

Heme oxygenase-1 and BVR, the latter responsible for the conversion of biliverdin into bilirubin, have been reported to exert beneficial effects in metabolic pathways. For instance, BVR has been shown to be a member of the insulin receptor substrate family (Lerner-Marmarosh et al., 2005) with pleiotropic functions that include effects on metabolism and cytoprotection (Kapitulnik and Maines, 2009). In addition, HO-1 has been shown to be induced by apoAI mimetic peptides and mediate some of their beneficial effects on decreasing endothelial cell sloughing and improving vascular reactivity in a rat model of diabetes (Kruger et al., 2005). Whether these effects are due to a systemic or a vascular level of action is not clear. HO-1 null mice do not exhibit alteration of plasma glucose and/or lipid levels indicative of overt diabetes, glucose intolerance, or other frank metabolic disorders. It might be possible that HO-1 deficient mice may experience upregulation of other compensatory mechanisms that could inhibit the development of obvious metabolic abnormalities.

In addition to these systemic effects, it also appears that vascular local expression is important. Indeed, adenoviral HO-1 overexpression via intracardiac administration, resulting in both aortic and liver overexpression, confers protection against atherosclerosis whereas tail vein administration, only resulting in hepatic but not aortic overexpression, does not (Juan et al., 2001). Although the contribution of systemic effects can not be ruled out by this approach since adenoviral administration via tail vein may have elicited a degree of systemic inflammation that could have masked the effects of sole hepatic HO-1 overexpression, the study does underline the importance of HO-1 expression in the vasculature.

### Endothelial cells

The vessels contain a monolayer of endothelial cells which represent the first cellular component that any infiltrating lipid or inflammatory cell encounter as they enter the vascular wall. The activation of endothelial cells leading to the expression of cell adhesion molecules (CAMs) and proinflammatory factors may be key in atherogenesis. Therefore, HO-1 expression in endothelial cells can be an important factor in the prevention against atherosclerosis. Indeed, Romanoski et al. (2011) recently reported that HO-1 expression in endothelial cells constitutes a critical gene in the global response of endothelial cells against oxidized phospholipids. In this study, a “systems-level” approach was used to understand the multiplicity of pathways that are elicited in HAECs, from 149 donors, in response to a treatment with oxPAPC, products of the oxidation of phospholipid PAPC (1-palmitoyl-2-arachidonyl-*sn*-glycero-3-phosphorylcholine), which are generated during the oxidation of LDL particles and known to induce HO-1. The approach took advantage of naturally occurring genetic variations in the human population that perturbed individual gene expression patterns with and without oxPAPC treatment, which enabled the construction of a gene co-expression network, consisting of 11 modules (groups) of tightly connected genes. Some genes exhibited many more connections than others (“hubs”), which in general, appear to be much more important in the functioning of the cell/organism as a whole. Notably, HO-1 emerged as a “hub” in one of the most interesting modules (blue module, Figure 3A), with various degrees of connection with other antioxidant genes (e.g. NQO1, Gclm), unfolded protein response (UPR) as well as transcription factors (e.g. Nrf2, MAFF).

This approach not only helped to identify novel factors that could be involved in the regulation of human HO-1 such as G-protein coupled receptor (GPR)-39 but also unraveled clues about the dynamics of the HO-1 response to oxPAPC in human subjects. While HAECs from the 149 donors exhibited a ninefold difference between extremes of HO-1 expression, they only exhibited a twofold difference after treatment with oxPAPC, suggesting that there was a ceiling to oxPAPC-induced HO-1 expression in this system. Importantly, basal HO-1 expression levels, rather than HO-1 levels after oxPAPC treatment, strongly correlated negatively with the expression of proinflammatory cytokines such as IL-6 (*r* = −0.36, *p* = 1.37 *e* − 06), IL-8 (*r* = −0.28, *p* = 2.41 *e* − 04), CAMs such as VCAM1 (*r* = −0.22, *p* = 3.31 *e* − 03), UPR such as Activating factor (ATF)3 (*r* = −0.40, *p* = 5.01*e* − 08). Silencing of HO-1 by siRNA resulted in upregulation of many of the same genes (Romanoski et al., 2011). Altogether, this suggests that basal HO-1 levels are major determinants in the protection against inflammation and possibly atherogenesis, in parallel with our similar findings in the models of liver ischemia reperfusion (Tsuchihashi et al., 2006) and cardiac allograft rejection (see Heme Oxygenase-1 and Vascular Inflammation: Importance of Basal Levels). In addition, it will be important to determine whether GPR39 or other genetic factors could help explain the ninefold difference observed in basal HO-1 in HAECs, especially since the (GT)n polymorphisms data could not explain that variation (Romanoski et al., 2011).

Heme oxygenase-1 expression in endothelial cells leads to decreased expression of vascular cell adhesion molecule-1 (VCAM-1) as well as the expression and release of chemokines and proinflammatory cytokines such as CC-chemokine ligand (CCL)2, also known as MCP-1 (Sacerdoti et al., 2005). Taha et al. also reported that HUVECs collected from individuals exhibited differences in their basal (unstimulated) HO-1 levels that were dependent on their genotype for the (GT)n microsatellite DNA. In this study, alleles were classified as Short (S), Medium (M), and Long (L) based on the number of GT repeats, ≤23, 24–28, and ≥29 repeats, respectively. Cells carrying the S allele exhibited higher HO-1 basal levels than those carrying the M and/or L alleles. Likewise, similar differences in expression levels ensued after treatment with H_2_O_2_, CoPP, LPS, 15d-PGJ_2b_, but not after treatment with hemin or in the presence of hypoxia where there were no differences. Here also, cells with the S allele, with higher basal HO-1 levels exhibited lower basal levels of IL1β, IL-6, and ICAM-1 (Taha et al., 2010). Despite the potential differences in behavior between endothelial cells in culture and those in the vascular wall *in vivo*, these studies support a protective role for HO-1 expression in endothelial cells that could represent a significant portion of its protection against atherogenesis.

Heme oxygenase-1 expression in endothelial cells could also play a role in the protection against atherosclerosis induced by environmental factors such as smoking and exposure to air pollution, which has been shown to lead to increased atherosclerosis in several animal models (Araujo, 2011). Indeed, it is upregulated in systemic tissues in response to the exposure to ambient ultrafine particles and its upregulation in a cell line of human microvascular endothelial cells, in response to oxPAPC and diesel exhaust particle (DEP) chemicals, occurs in a synergistic manner (Gong et al., 2007). It remains to be determined whether HO-1 plays indeed a protective role in this context.

### Macrophages, dendritic cells, and lymphocytes

**Figure 5:**
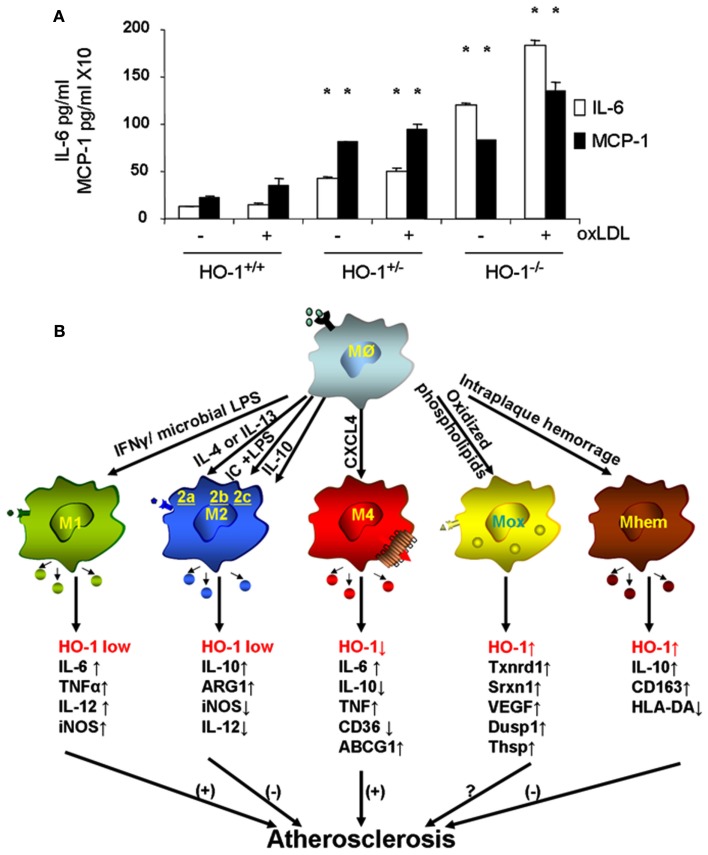
**Heme oxygenase-1 inhibits macrophage proinflammatory activity**. **(A)** Peritoneal macrophages from HO-1^+/+^, HO-1^+/−^, and HO-1^+/+^ mice were cultured in the presence or absence of oxLDL 50 μg/ml for 6 h. IL-6 and MCP-1 were determined by ELISA. *p < 0.001 as compared with controls. Data taken from Orozco et al. (2007). **(B)** HO-1 expression varies in different subtypes of macrophages. Upon monocyte differentiation into macrophages, they can polarize into one of the five proposed subtypes, M1, M2, M4, Mox, and Mhem. Each subtype is induced by specific stimuli as shown along the arrows that derive from the macrophage Mø atop, and characterized by a set of phenotypic markers and/or gene expression shown below each one of them. While some of macrophage subtypes are proposed to simulate atherogenesis such as M1 and M4, others are proposed to inhibit lesion development such as M2 and Mhem. Mox macrophages, generated after treatment with oxPAPC, are dependent on Nrf2 with a role in atherogenesis still to be determined.

Macrophages are the main inflammatory cells that infiltrate the vascular wall in atherogenesis, involved in the initiation as well as progression of atherosclerotic lesions. Upon activation of endothelial cells, blood monocytes are recruited to the site of injury and/or EC activation where cytokines and/or chemokines are released, adhere to the endothelium and transmigrate to the subendothelial space where they further differentiate into macrophages. HO-1 is highly upregulated in macrophages in atherosclerotic lesions and several lines of evidence support its protective antiatherogenic role in these cells. First, HO-1 has been shown to exert antioxidant and antiinflammatory effects in macrophages (Lee et al., 2003; Philippidis et al., 2004; Levonen et al., 2007; Orozco et al., 2007). Indeed, we have reported that decreased or absent HO-1 expression in peritoneal macrophages results in enhanced ROS formation and increased inflammatory cytokines such as MCP-1, interleukin 6 (IL-6), and the murine interleukin 8 homolog (KC; Orozco et al., 2007; Figure 5A). Second, HO-1 expression influences lipid loading and foam cell formation as both partial and total HO-1 deficiency results in enhanced lipid loading and foam cell formation in peritoneal macrophages treated with oxLDL, at least partially mediated by increased expression of the scavenger receptor A (SR-A; Orozco et al., 2007). Third, lack of HO-1 expression in bone marrow-derived cells resulted in atherosclerotic plaques with a larger inflammatory component, suggestive of greater vulnerability for rupture (Orozco et al., 2007). Irradiated LDL-R^−/−^ mice with their bone marrow reconstituted with HO-1^−/−^ donor cells resulted in plaques with increased macrophage area as compared with control mice reconstituted with WT bone marrow (Orozco et al., 2007). Fourth, HO-1 expression may play a role in macrophage differentiation and polarization (Figure 5B).

Heme oxygenase-1 antiatherogenic actions may also be due to its expression in other myelocytic or lymphocytic cells. Thus, it has been shown that HO-1 is expressed in immature dendritic cells and its overexpression inhibits dentritic cell maturation (Chauveau et al., 2005) which may play a role in atherogenesis. Several studies have highlighted a prominent role of dendritic cells in atherosclerotic lesions (Llodra et al., 2004; Yilmaz et al., 2004; Bobryshev, 2005) as they are present especially in regions of atherosclerotic plaques that are prone to rupture (Yilmaz et al., 2004) and its emigration from the lesions to lymph nodes associate with reduction in the size of atherosclerotic plaques (Llodra et al., 2004). Antigen presentation by dendritic cells as well as lymphocyte infiltration and the triggering of Th1 immune responses appear to play a role in atherosclerosis (Bobryshev, 2010; Manthey and Zernecke, 2011). Atherosclerotic lesion development is favored by Th1 responses while it is inhibited by conditions that promote a Th2 response (Minamino et al., 2001). HO-1 overexpression may lead to a switch from a Th1 to a Th2 response (Ke et al., 2000, 2001). These immunomodulatory actions parallel the effects of HO-1 expression against acute rejection response in models of allograft transplantation (Araujo et al., 2003). As macrophages can also function as antigen-presenting cells, they can play a role in the modulation of Th immune responses. Therefore, macrophage HO-1 expression could not only affect their function as effectors but also as presenting cells.

Macrophage differentiation, polarization, and function could indeed be affected by HO-1 expression. Upon differentiation, macrophages may polarize into distinct subsets with characteristic phenotypes and likely to exhibit different behaviors in atherosclerotic lesion formation, as schematized in Figure 5B. The first subtypes of polarized macrophages that were described were named as M1 and M2. Macrophages stimulated by lipopolysaccharide (LPS) or interferon gamma (IFN-γ) polarize into M1 macrophages, characterized by increased expression of proinflammatory molecules such as IL-1β, IL-6, IL-8, tumor necrosis factor alpha (TNF-α) as well as inducible nitric oxide synthase (iNOS), which typically trigger Th1 T cell responses (Wuttge et al., 2001; Yan and Hansson, 2007; Krausgruber et al., 2011). On the other hand, M2 macrophages stimulated by IL-4 (M2a), immune complexes (M2b), or IL-10/IL-13 (M2c) are characterized by increased expression of antiinflammatory molecules including IL-10, arginase-1 (Arg1), mannose receptor, tumor growth factor beta (TGF-β), which are likely to play an antiatherosclerotic role (Wilson, 2010). While both types of macrophages have been identified in atherosclerotic lesions (Bouhlel et al., 2007) and exhibit low expression of HO-1, it has been reported that increased HO-1 expression may contribute to a M2 macrophage activation profile, which is involved in the resolution of inflammation (Weis et al., 2009). Furthermore, different macrophage subtypes have been recently described where expression of HO-1 may play a characteristic role, either as a part of their phenotypic traits or in the modulation of their potential role in lesion formation.

Treatment of macrophages with oxPAPC led to a phenotypic switch of both M1 and M2 phenotypes into a new subtype named as Mox, characterized by exclusive upregulation of redox-regulated genes such as HO-1, sulforedoxin-1 (Srxn1), glutamate-cysteine ligase, modifier subunit (Gclm), glutamate-cysteine ligase, catalytic subunit (Gclc), thioredoxin reductase (Txnrd) 1, but also vascular endothelial growth factor (VEGF), nuclear receptor Nr4A2 (Nurr1) and Trb3 (Kadl et al., 2010). Approximately 30% of macrophages present in atherosclerotic lesions developed by LDLR^−/−^ mice had this phenotype. Interestingly, polarization into a Mox phenotype was Nrf2 dependent and also characterized by a decreased phagocytic activity (Kadl et al., 2010), which raises the question whether this macrophage could exert proatherosclerotic actions, despite its high expression of HO-1 and other antioxidant genes (Araujo, 2012). More recently, another subtype of macrophages has been described in association with intraplaque hemorrhage named as Mhem, characterized by increased expression of IL-10 and CD163, macrophage scavenge receptor for hemoglobin-haptoglobin complex (Hb-Hp), low expression of human leukocyte antigen-DR (HLA-DR; Boyle et al., 2009). Mhem macrophages also exhibit increased expression of HO-1, dependent on Nrf2 (Boyle et al., 2011) in addition to activating transcription factor 1 (Boyle et al., 2012). These macrophages could play an antiatherogenic role by virtue of its participation in Hb-Hp clearance, with subsequent reduction in ROS formation and antiinflammatory effects. One last macrophage subtype named M4 is induced by CXCL4 and characterized by a gene expression profile that contained both M1 and M2 genes (Gleissner et al., 2010). Unlike Mhem macrophages which have increased expression of CD163 (also known as ED2) and HO-1, M4 macrophages do not express Hb-Hp scavenger receptor CD163 and do not upregulate HO-1 expression (Gleissner, 2012). M4 macrophages appear to play a proatherogenic role, which is consistent with the ~60% decrease in aortic atherosclerotic lesions exhibited by CXCL-4^−/−^ApoE^−/−^ mice (Sachais et al., 2007). It is clear then that macrophage differentiation and polarization is quite complex as they may respond to the very specific microenvironments that monocytes and macrophages encounter. These various macrophage subtypes exhibit different patterns of HO-1 expression (Figure 5B) and it is unknown whether HO-1 could play a role in the polarization into any of these subtypes or in the function that these macrophages could have in atherogenesis. For instance, Nrf2 expression appears to be essential in the generation of Mox macrophages and participate in the generation of Mhem macrophages as well, both of which express high levels of HO-1 but perhaps with different roles in the development of atherosclerotic lesions. Thus, the role for HO-1 expression in these various macrophage subtypes still need to be determined (Figure 5B).

### Smooth muscle cells

Heme oxygenase-1 is also expressed in vascular SMCs and could play an important role in the protection against atherogenesis. This is supported by evidence accrued in the model of post-angioplasty restenosis where the proliferative response of SMCs constitutes a fundamental aspect of the disease (Duckers et al., 2001; Tulis et al., 2001). SMC proliferative response has been shown to be important in the progression of atherosclerotic plaques as well. On the other hand, SMC and their synthesis of collagen are important contributors to plaque composition and stability as thicker fibrous caps are associated with lesser vulnerability for rupture. As mentioned above, inhibition of HO activity by ZnPPIX decreased relative cap thickness and intimal surface of SMC in ApoE null mice (Cheng et al., 2009). Likewise, administration of SnPPIX to rabbits resulted in plaques with decreased content of SMC while hemin led to the opposite (Li et al., 2011). Whether these effects are due to HO-1 expression in SMC *per se* or due its expression in neighboring cells is not clear.

In summary, the overall antiatherogenic role of HO-1 expression may be due to a combination of complex systemic and vascular local effects that converge in the inhibition of lipid peroxidation with effects on circulating lipoproteins, decrease in the activation of endothelial cells, inhibition of macrophage proinflammatory activity and possible participation in its differentiation/polarization, inhibition of dendritic cell maturation as well as modulation of immune-mediated responses and regulation of the proliferative response of SMCs.

## Heme Oxygenase System, Enzymatic Byproducts, and Protection Against Atherosclerosis

Heme oxygenase-1 is not only expressed ubiquitously in the body but it is also located in several compartments within the cell. It is a 32-kDa protein most abundantly located in the microsomal fraction, with a short transmembrane segment of ~ 2 kDa in the endoplasmic reticulum membrane and ~30 kDa cytosolic portion. However, HO-1 can also be found in the plasma membrane, associated with caveolae (Kim et al., 2004), in the mitochondria (Converso et al., 2006) and in the nucleus (Lin et al., 2007, 2008). Interestingly, while nuclear HO-1 was shown to be catalytically inactive (Lin et al., 2008), it appeared to modulate the expression of itself (Lin et al., 2008) and that of activating protein (AP)-1 (Lin et al., 2007). Although HO-1 does not have DNA binding domains in its sequence and no definite protein-protein interactions have been demonstrated, additional work is required to elucidate whether HO-1 may play a role in transcriptional regulation. It appears that the biological effects due to HO-1 expression are mostly due to its enzymatic activity, since its pharmacological inhibition results in almost complete abolishment of those biological effects. Therefore, the various byproducts of HO enzymatic activity could be considered as its “branches” or “arms of action.”

### Biliverdin/bilirubin

There is evidence that biliverdin/bilirubin can mediate some of HO-1 antiatherogenic effects. Exogenous administration of biliverdin, which is promptly converted to bilirubin, appears to be effective in inhibiting atherogenesis in ApoE null mice (personal communication from Miguel Soares, Gulbenkian Institute of Sciences, Portugal). This parallels the protective effects of biliverdin and/or bilirubin in other models of vascular inflammation, such as liver ischemia reperfusion injury (Fondevila et al., 2004), cardiac allograft graft rejection (Yamashita et al., 2004), and post-angioplasty restenosis (Ollinger et al., 2005, 2007). In addition, epidemiological studies show an inverse relationship of plasma or serum bilirubin concentrations and the risk of CAD (Schwertner et al., 1994; Schwertner and Vitek, 2008), at a strength that was found to be similar to that of smoking, elevated systolic blood pressure, and low HDL cholesterol (Schwertner et al., 1994; Hopkins et al., 1996; Schwertner, 1998). Serum bilirubin concentrations are inversely correlated with the severity of atherosclerosis in men (Novotny and Vitek, 2003). Thus, individuals with Gilbert syndrome, characterized by increased levels of circulating unconjugated bilirubin due to a low reactivity of the bilirubin-uridine diphosphate glucuronyl transferase (bilirubin-UGT) enzyme, exhibit a marked reduction in CHD risk (Schwertner and Vitek, 2008).

Biliverdin/Biliribin could inhibit atherogenesis through to its antioxidant properties. Biliverdin’s antioxidant properties appear to depend on the expression of BVR (Singleton and Laster, 1965) and its conversion to bilirubin. Indeed, bilirubin is a powerful antioxidant (Stocker et al., 1987; Stocker and Keaney, 2004) that could inhibit the oxidation of LDL and other lipids (Neuzil and Stocker, 1994; Wu et al., 1996), scavenge oxygen radicals (Stocker et al., 1987) and counteract oxidative stress overall (Schwertner, 1998). The production of bilirubin could participate in an amplification cycle by getting oxidized into biliverdin and then reduced back into bilirubin by BVR (Baranano et al., 2002).

Bilirubin also exerts antiinflammatory and antiproliferative actions that can account for its antiatherogenic effects. Indeed, congenitally hperbilirubinemic Gunn rats exhibit almost no neointimal proliferation after balloon injury in comparison with significant proliferation observed in WT rat controls (Ollinger et al., 2005). Likewise, 1-h local treatment of a rat carotid artery with biliverdin significantly reduces neointima formation after balloon injury (Ollinger et al., 2007). These effects were mediated by inhibition of cell cycle progression and proliferation of SMC (Ollinger et al., 2005, 2007), which could protect against atherosclerosis (Sriram and Patterson, 2001). Various mechanisms may explain antiproliferative effects of Biliverdin/Bilirubin such as: (1) Overexpression of cdk inhibitor p53 resulting in increased apoptosis of rat SMCs (Liu et al., 2002), (2) Inhibition of cyclin A, D1 and E, YY1 and cdk 2 expression, leading to hypophosphorylation of the retinoblastoma tumor suppressor protein (Rb) and cell cycle arrest (Ollinger et al., 2005), (3) Inhibition of JNK activation (Ollinger et al., 2007), and (4) Modulation of p38 MAPK activation (Ollinger et al., 2005). Interestingly, bilirubin/biliverdin have been shown to be ligands of the aryl hydrocarbon receptor (Ahr) in mouse hepatoma hepa 1c1c7 cells (Sinal and Bend, 1997; Phelan et al., 1998) and SMCs from Ahr KO mouse aortas exhibit a prolonged cell cycle time as compared with controls (Ollinger et al., 2007), which suggest that Ahr activation could participate in the regulation of proliferation of SMCs. In support of this possibility, Ahr null mice exhibit decreased atherosclerosis in the ApoE null background (Wu et al., 2011).

All this evidence, together with the findings that relate the participation of BVR in insulin-induced pathways (Lerner-Marmarosh et al., 2005; Kapitulnik and Maines, 2009), make this arm attractive candidate for pharmacological intervention. Thus, development of compounds that either increase the endogenous levels of biliverdin/bilirubin by targeting genes involved in the metabolism or resemble their biological activity can lead to promising new therapies.

### Carbon monoxide

The generation of CO constitutes the second arm how HO-1 may prevent atherosclerosis. In fact, administration of CO by inhalation results in suppression of intimal hyperplasia associated with chronic graft rejection and balloon injury (Otterbein et al., 2003). Although there are no reports yet about CO inhibitory effects on atherosclerotic lesions due to hyperlipidemia, there are several lines of evidence and potential mechanisms that make it very likely. First, CO exhibits cytoprotective properties in vascular endothelial cells by inhibiting apoptosis, likely through activation of p38 mitogen-activated protein kinase (MAPK) signaling pathway (Brouard et al., 2000). Second, CO exerts antiinflammatory effects. Indeed, CO has been shown to modulate the response of monocytes/macrophages to bacterial LPS (Otterbein et al., 2000), decrease macrophage toll-like receptor (TLR) signaling with downstream activation of NF-kB (Wang et al., 2009) and inhibit expression of GM-CSF with probable alteration of macrophage differentiation (Sarady et al., 2002). Inhalation of CO leads to decreased leukocyte infiltration of rat aortic allografts (Otterbein et al., 2003), accompanied by decreased expression of proinflammatory genes associated with macrophage activation (Otterbein et al., 2000). Interestingly, antiinflammatory effects are also mediated by activation of the MAPK signaling pathway, but they appear to require the generation of ROS as inhibition of ROS in macrophages leads to loss of CO antiinflammatory effects (Bilban et al., 2008). Third, HO-1 and CO may crosstalk with Nitric Oxide Synthases (NOS) and NO. Administration of hemin to rabbits resulted in greater CO levels in the aorta, accompanied by decreased levels of aortic NO and inducible Nitric oxide synthase (iNOS; Li et al., 2011), while HO inhibition by SnPPIX or ZnPPIX resulted in the opposite (Li et al., 2011; Liu et al., 2012). CO might bind and activate guanylate cyclase, leading to increased intracellular levels of cyclic guanine monophosphate (cGMP; Morita et al., 1995; Hartsfield, 2002). However, the end result in this crosstalk between CO and NO may depend on their relative concentrations as both gaseous molecules compete for the same enzyme but with different affinities. In addition, the biological effects due to this interplay between CO and NO may be characteristic of each cell type. Fourth, CO displays antiproliferative actions in SMCs (Morita et al., 1997), which could account for a good portion of its beneficial effects in the model of balloon injury (Otterbein et al., 2003). These CO effects require activation of p38 MAPK pathway, which appears to occur via a cGMP-dependent mechanism and require activation of guanylate cyclase as well (Otterbein et al., 2003). Last, there is a possibility that CO could exert antioxidant effects via binding of the Fe^2+^ in heme groups and preventing the oxidation of hemoproteins, subsequently inhibiting the release of free heme (Balla et al., 1993). This mechanism appears to be important in the suppression of experimental cerebral malaria by CO (Pamplona et al., 2007).

### Heme catabolism and release of iron

A third arm of action of the HO-1 system with antioxidant effects consists in the catabolic processing of heme groups, including the release and safe disposal of ferrous iron (Fe^2+^). Heme groups, derived from hemoglobin and other hemoproteins, can exacerbate the generation of ROS and membrane lipid peroxidation via the participation of Fe^2+^ in the Fenton reaction (Ryter and Tyrrell, 2000). Heme groups consist of a central iron atom in a coordinated binding to a protoporphyrin, composed of four pyrrole rings. The oxidative cleavage of this tetrapyrrolic ring leads to the release of its central Fe^2+^ with the potential of facilitating its participation in redox chemistry. This is how in certain circumstances, overexpression of HO, either HO-1 or HO-2 have been shown to exert prooxidant effects instead (Lamb et al., 1999; Ryter and Tyrrell, 2000). However, the simultaneous expression of ferritin, an important multimeric protein complex with a high capacity for iron storage, helps to channel the safe disposal for Fe^2+^. In addition, it appears that several of the same stimuli that trigger HO-1 upregulation also increase ferritin expression, at the same time that labile Fe *per se* can upregulate the expression of heavy-chain (H-) ferritin, which in association with light-chain (L-) ferritin subunits lead to the assembly of ferritin protein (Harrison and Arosio, 1996). Indeed, ferritin expression colocalizes with HO-1 expression in atherosclerotic lesions and in common with HO-1, ferritin is markedly increased in various cell types present in atherosclerotic plaques such as myofibroblasts, macrophages, and endothelial cells, where it may play a protective role against oxidized lipoproteins (Juckett et al., 1995). Coordinate expression of HO-1 and ferritin can lead to antioxidant effects by different mechanisms. First, it decreases the bioavailability of Fe^2+^ to participate in redox chemistry. Second, the ferroxidase activity of the H-chain subunit, leads to the conversion of Fe^2+^ to Fe^3+^ and therefore, decrease in iron prooxidant potential (Balla et al., 1992; Berberat et al., 2003). Third, multimeric ferritin can bind free heme with subsequent reduction in its bioavailability (Kadir and Moore, 1990). The prooxidative effects of HO-1 overexpression could be seen when increases in HO-1 levels overwhelm concurrent expression of ferritin. It seems, however, that these potentially deleterious effects that are noted in experimental studies are less likely to occur in normal physiological conditions. Instead, co-expression of both HO and ferritin appears to play a significant antioxidant role.

Overall, it appears that all HO byproducts could contribute to the HO-1 antiatherogenic effects. It remains to be elucidated whether these actions are complementary or merely overlapping and what is the degree of contribution of each one of these arms of action in the HO-1 global effects.

## Summary and Perspectives

The overall importance of HO is underlined by its large degree of evolutionary conservation in all kingdoms of life as it is expressed in prokaryotic and eukaryotic cells, bacteria, yeasts, protozoa, plants, invertebrate and vertebrate animals. In all these various living organisms, HO contributes to the catabolic processing of hemoproteins and avoidance of the potential toxicity that could be associated to the release of Fe atoms present on them.

Heme oxygenase appears to exert most of its actions thanks to its enzymatic activity and the generation of various enzymatic byproducts that act on a multiplicity of pathways. Therefore, HO activity can be visualized as a HO system with three branches or arms of action that consist in: (1) generation of Biliverdin and subsequent reduction to bilirubin, (2) release of CO, and (3) release and safe disposal of Fe^2+^. These arms of action modulate various pathways with a high degree of overlap and/or cross-over, which may help to ensure the induction of the desired biological effects. HO-1 is most abundant in the cytosol, attached to the smooth endoplasmic reticulum (SER) membrane. However, its presence in other subcellular localizations such as the plasma membrane, mitochondria, or nucleus raises the question whether HO could exert some protein-protein or protein-DNA interactions that could play other roles, such as transcriptional regulation, which requires further exploration. HO-1 and the HO system in general are thought to play a critical role in cellular homeostasis where the presence of complementarity or overlap between the arms of action could result in some degree of redundancy that can help to ensure the induction of HO mediated critical effects. These principles are also seen in the protection against vascular inflammation and atherosclerosis. Thus, HO three arms of action have various degrees of antioxidant, antiinflammatory, antiapoptotic, antiproliferative, and immunomodulatory effects, most of which may play a significant role in the protection against atherosclerotic lesion formation.

Heme oxygenase-1 has been shown to be a critical gene in the cellular response against prooxidative stimuli such as oxidized phospholipids. When considering the cellular response as a whole, HO-1 acts as a hub of gene-gene interactions that modulate the triggering and activation of several protective molecular pathways. This has been demonstrated in endothelial cells and is likely to be the case in other vascular cells such as monoctyes/macrophages. In addition, HO-1 may be a protective gene against proatherogenic effects caused by environmental factors such as cigarette smoking or exposure to air pollution.

Basal HO-1 mRNA expression levels in response to injurious stimuli appear to be more important than the degree of upregulation or fold induction for the protection against those stimuli in the vasculature. In some circumstances, the levels of HO-1 protein and HO functional activity may not be concordant which could be due to posttranslational modifications. Therefore, a better understanding of their kinetics in vascular processes such as atherosclerosis is required to shed light on the reasons why very high levels of upregulated HO-1 in vascular cells may be overwhelmed by the pathogenic process and fail to abrogate the progression of atherosclerotic lesions. Importantly, HO-1 can be targeted pharmacologically by interventions that seek its upregulation or enhancement of its various arms of action, via: (1) induction of HO-1 expression with drugs such as heme arginate, (2) administration of CO by inhalation or use of CO releasing molecules (CORM), and (3) administration of biliverdin/bilirubin or use of inhibitors of bilirubin conjugation that would result in increased bilirubin levels.

One potential caveat, however, is that modulation of HO-1 could result in alteration of the activation status of its transcription factor Nrf2, which has been shown to exert proatherosclerotic effects. The discordant effects between Nrf2 and HO-1 underline the high degree of complexity that therapeutic modulation of vascular oxidative stress must address. This is in addition to studies that have shown that while some oxidation products promote inflammation in the vascular wall, others could have antiinflammatory activity instead. Indeed, electrophilic nitro-fatty acids which are formed via nitric oxide or nitrite-dependent redox reactions have been shown to exert antiinflammatory actions (Khoo and Freeman, 2010). Therefore, any pharmacological modulation of HO-1 should take into consideration any potential alteration of the Nrf2 activation status.

## Conflict of Interest Statement

The authors declare that the research was conducted in the absence of any commercial or financial relationships that could be construed as a potential conflict of interest.

## References

[B1] Abraham N. G. Kappas A. (2008). Pharmacological and clinical aspects of heme oxygenase. Pharmacol. Rev. 60, 79–127 10.1124/pr.107.07104 18323402

[B2] Amersi F. Buelow R. Kato H. Ke B. Coito A. J. Shen X. D. Zhao D. Zaky J. Melinek J. Lassman C. R. Kolls J. K. Alam J. Ritter T. Volk H. D. Farmer D. G. Ghobrial R. M. Busuttil R. W. Kupiec-Weglinski J. W. (1999). Upregulation of heme oxygenase-1 protects genetically fat Zucker rat livers from ischemia/reperfusion injury. J. Clin. Invest. 104, 1631–1639 10.1172/JCI7903 10587527 PMC409865

[B3] Araujo J. (2011). Particulate air pollution, systemic oxidative stress, inflammation, and atherosclerosis. Air Qual. Atmos. Health 4, 79–93 10.1007/s11869-010-0101-8 21461032 PMC3040314

[B4] Araujo J. Ishikawa K. Lusis A. J. (2002). “Heme Oxygenase and Atherosclerosis,” in Heme Oxygenase: Biology and Medicine, ed. Abraham N. (New York: Kluwer Publisher), 269–278

[B5] Araujo J. A. (2012). Nrf2 and the promotion of atherosclerosis: lessons to be learned. Clin. Lipidol. 7, 123–116 10.2217/clp.12.5

[B6] Araujo J. A. Meng L. Tward A. D. Hancock W. W. Zhai Y. Lee A. Ishikawa K. Iyer S. Buelow R. Busuttil R. W. Shih D. M. Lusis A. J. Kupiec-Weglinski J. W. (2003). Systemic rather than local heme oxygenase-1 overexpression improves cardiac allograft outcomes in a new transgenic mouse. J. Immunol. 171, 1572–1580 12874251 10.4049/jimmunol.171.3.1572

[B7] Balla G. Jacob H. S. Balla J. Rosenberg M. Nath K. Apple F. Eaton J. W. Vercellotti G. M. (1992). Ferritin: a cytoprotective antioxidant strategem of endothelium. J. Biol. Chem. 267, 18148–18153 1517245

[B8] Balla G. Jacob H. S. Eaton J. W. Belcher J. D. Vercellotti G. M. (1991). Hemin: a possible physiological mediator of low density lipoprotein oxidation and endothelial injury. Arterioscler. Thromb. Vasc. Biol. 11, 1700–1711 10.1161/01.ATV.11.6.1700 1931871

[B9] Balla J. Jacob H. S. Balla G. Nath K. Eaton J. W. Vercellotti G. M. (1993). Endothelial-cell heme uptake from heme proteins: induction of sensitization and desensitization to oxidant damage. Proc. Natl. Acad. Sci. U.S.A. 90, 9285–9289 10.1073/pnas.90.20.9285 8415693 PMC47552

[B10] Barajas B. Che N. Yin F. Rowshanrad A. Orozco L. D. Gong K. W. Wang X. Castellani L. W. Reue K. Lusis A. J. Araujo J. A. (2011). NF-E2-related factor 2 promotes atherosclerosis by effects on plasma lipoproteins and cholesterol transport that overshadow antioxidant protection. Arterioscler. Thromb. Vasc. Biol. 31, 58–66 10.1161/ATVBAHA.110.210906 20947826 PMC3037185

[B11] Baranano D. E. Rao M. Ferris C. D. Snyder S. H. (2002). Biliverdin reductase: a major physiologic cytoprotectant. Proc. Natl. Acad. Sci. U.S.A. 99, 16093–16098 10.1073/pnas.252626999 12456881 PMC138570

[B12] Barry-Lane P. A. Patterson C. Van Der Merwe M. Hu Z. Holland S. M. Yeh E. T. Runge M. S. (2001). p47phox is required for atherosclerotic lesion progression in ApoE(-/-) mice. J. Clin. Invest. 108, 1513–1522 10.1172/JCI200111927 11714743 PMC209414

[B13] Berberat P. O. Katori M. Kaczmarek E. Anselmo D. Lassman C. Ke B. Shen X. Busuttil R. W. Yamashita K. Csizmadia E. Tyagi S. Otterbein L. E. Brouard S. Tobiasch E. Bach F. H. Kupiec-Weglinski J. W. Soares M. P. (2003). Heavy chain ferritin acts as an antiapoptotic gene that protects livers from ischemia reperfusion injury. FASEB J. 17, 1724–1726 12958189 10.1096/fj.03-0229fje

[B14] Bilban M. Haschemi A. Wegiel B. Chin B. Y. Wagner O. Otterbein L. E. (2008). Heme oxygenase and carbon monoxide initiate homeostatic signaling. J. Mol. Med. 86, 267–279 10.1007/s00109-007-0276-0 18034222

[B15] Bobryshev Y. V. (2005). Dendritic cells in atherosclerosis: current status of the problem and clinical relevance. Eur. Heart J. 26, 1700–1704 10.1093/eurheartj/ehi282 15855191

[B16] Bobryshev Y. V. (2010). Dendritic cells and their role in atherogenesis. Lab. Investig. 90, 970–984 10.1038/labinvest.2010.94 20458277

[B17] Bouhlel M. A. Derudas B. Rigamonti E. Dievart R. Brozek J. Haulon S. Zawadzki C. Jude B. Torpier G. Marx N. Staels B. Chinetti-Gbaguidi G. (2007). PPAR gamma activation primes human monocytes into alternative M2 macrophages with anti-inflammatory properties. Cell Metab. 6, 137–143 10.1016/j.cmet.2007.06.010 17681149

[B18] Boyle J. J. Harrington H. A. Piper E. Elderfield K. Stark J. Landis R. C. Haskard D. O. (2009). Coronary intraplaque hemorrhage evokes a novel atheroprotective macrophage phenotype. Am. J. Pathol. 174, 1097–1108 10.2353/ajpath.2009.080431 19234137 PMC2665768

[B19] Boyle J. J. Johns M. Kampfer T. Nguyen A. T. Game L. Schaer D. J. Mason J. C. Haskard D. O. (2012). Activating transcription factor 1 directs mhem atheroprotective macrophages through coordinated iron handling and foam cell protection. Circ. Res. 110, 20–33 10.1161/CIRCRESAHA.111.247577 22052915

[B20] Boyle J. J. Johns M. Lo J. Chiodini A. Ambrose N. Evans P. C. Mason J. C. Haskard D. O. (2011). Heme induces heme oxygenase 1 via Nrf2 role in the homeostatic macrophage response to intraplaque hemorrhage. Arterioscler. Thromb. Vasc. Biol. 31, U2685–U2826 10.1161/ATVBAHA.111.225813 21868703

[B21] Brasen J. H. Leppanen O. Inkala M. Heikura T. Levin M. Ahrens F. Rutanen J. Pietsch H. Bergqvist D. Levonen A. L. Basu S. Zeller T. Kloppel G. Laukkanen M. O. Yla-Herttuala S. (2007). Extracellular superoxide dismutase accelerates endothelial recovery and inhibits in-stent restenosis in stented atherosclerotic Watanabe heritable hyperlipidemic rabbit aorta. J. Am. Coll. Cardiol. 50, 2249–2253 10.1016/j.jacc.2007.08.038 18061074

[B22] Brouard S. Otterbein L. E. Anrather J. Tobiasch E. Bach F. H. Choi A. M. Soares M. P. (2000). Carbon monoxide generated by heme oxygenase 1 suppresses endothelial cell apoptosis. J. Exp. Med. 192, 1015–1026 10.1084/jem.192.7.1015 11015442 PMC2193315

[B23] Burns M. J. Furie M. B. (1998). Borrelia burgdorferi and interleukin-1 promote the transendothelial migration of monocytes in vitro by different mechanisms. Infect. Immun. 66, 4875–4883 9746592 10.1128/iai.66.10.4875-4883.1998 PMC108603

[B24] Callegari A. Liu Y. White C. C. Chait A. Gough P. Raines E. W. Cox D. Kavanagh T. J. Rosenfeld M. E. (2011). Gain and loss of function for glutathione synthesis: impact on advanced atherosclerosis in apolipoprotein E-deficient mice. Arterioscler. Thromb. Vasc. Biol. 31, 2473–2482 10.1161/ATVBAHA.111.229765 21868708 PMC3415243

[B25] Chauveau C. Remy S. Royer P. J. Hill M. Tanguy-Royer S. Hubert F. X. Tesson L. Brion R. Beriou G. Gregoire M. Josien R. Cuturi M. C. Anegon I. (2005). Heme oxygenase-1 expression inhibits dendritic cell maturation and proinflammatory function but conserves IL-10 expression. Blood 106, 1694–1702 10.1182/blood-2005-02-0494 15920011

[B26] Chen Y. H. Chau L. Y. Lin M. W. Chen L. C. Yo M. H. Chen J. W. Lin S. J. (2004). Heme oxygenase-1 gene promotor microsatellite polymorphism is associated with angiographic restenosis after coronary stenting. Eur. Heart J. 25, 39–47 10.1016/j.ehj.2003.10.009 14683741

[B27] Chen Y. H. Lin S. J. Lin M. W. Tsai H. L. Kuo S. S. Chen J. W. Charng M. J. Wu T. C. Chen L. C. Ding Y. A. Pan W. H. Jou Y. S. Chau L. Y. (2002). Microsatellite polymorphism in promoter of heme oxygenase-1 gene is associated with susceptibility to coronary artery disease in type 2 diabetic patients. Hum. Genet. 111, 1–8 10.1007/s00439-002-0769-4 12136229

[B28] Cheng C. Noordeloos A. M. Jeney V. Soares M. P. Moll F. Pasterkamp G. Serruys P. W. Duckers H. J. (2009). Heme oxygenase 1 determines atherosclerotic lesion progression into a vulnerable plaque. Circulation 119, 3017–3027 10.1161/CIRCULATIONAHA.108.814996 19487598

[B29] Choi A. M. Alam J. (1996). Heme oxygenase-1: function, regulation, and implication of a novel stress-inducible protein in oxidant-induced lung injury. Am. J. Respir. Cell Mol. Biol. 15, 9–19 8679227 10.1165/ajrcmb.15.1.8679227

[B30] Coito A. J. Buelow R. Shen X. D. Amersi F. Moore C. Volk H. D. Busuttil R. W. Kupiec-Weglinski J. W. (2002). Heme oxygenase-1 gene transfer inhibits inducible nitric oxide synthase expression and protects genetically fat Zucker rat livers from ischemia-reperfusion injury. Transplantation 74, 96–102 10.1097/00007890-200207150-00017 12134106

[B31] Converso D. P. Taille C. Carreras M. C. Jaitovich A. Poderoso J. J. Boczkowski J. (2006). HO-1 is located in liver mitochondria and modulates mitochondrial heme content and metabolism. FASEB J. 20, 1236–1238 10.1096/fj.05-4204fje 16672635

[B32] Duckers H. J. Boehm M. True A. L. Yet S. F. San H. Park J. L. Clinton Webb R. Lee M. E. Nabel G. J. Nabel E. G. (2001). Heme oxygenase-1 protects against vascular constriction and proliferation. Nat. Med. 7, 693–698 10.1038/89068 11385506

[B33] Durand E. Al Haj Zen A. Addad F. Brasselet C. Caligiuri G. Vinchon F. Lemarchand P. Desnos M. Bruneval P. Lafont A. (2005). Adenovirus-mediated gene transfer of superoxide dismutase and catalase decreases restenosis after balloon angioplasty. J. Vasc. Res. 42, 255–265 10.1159/000085658 15870505

[B34] Durante W. (2011). Protective role of heme oxygenase-1 against inflammation in atherosclerosis. Front. Biosci. 16, 2372–2388 10.2741/3860 21622183 PMC5940339

[B35] Exner M. Schillinger M. Minar E. Mlekusch W. Schlerka G. Haumer M. Mannhalter C. Wagner O. (2001). Heme oxygenase-1 gene promoter microsatellite polymorphism is associated with restenosis after percutaneous transluminal angioplasty. J. Endovasc. Ther. 8, 433–440 10.1583/1545-1550(2001)008<0433:HOGPMP>2.0.CO;2 11718398

[B36] Fondevila C. Shen X. D. Tsuchiyashi S. Yamashita K. Csizmadia E. Lassman C. Busuttil R. W. Kupiec-Weglinski J. W. Bach F. H. (2004). Biliverdin therapy protects rat livers from ischemia and reperfusion injury. Hepatology 40, 1333–1341 10.1002/hep.20480 15565657

[B37] Freigang S. Ampenberger F. Spohn G. Heer S. Shamshiev A. T. Kisielow J. Hersberger M. Yamamoto M. Bachmann M. F. Kopf M. (2011). Nrf2 is essential for cholesterol crystal-induced inflammasome activation and exacerbation of atherosclerosis. Eur. J. Immunol. 41, 2040–2051 10.1002/eji.201190044 21484785

[B38] Gleissner C. A. (2012). Macrophage phenotype modulation by CXCL4 in atherosclerosis. Front. Physiol. 3:1 10.3389/fphys.2012.00001 22275902 PMC3257836

[B39] Gleissner C. A. Shaked I. Little K. M. Ley K. (2010). CXC chemokine ligand 4 induces a unique transcriptome in monocyte-derived macrophages. J. Immunol. 184, 4810–4818 10.4049/jimmunol.0901368 20335529 PMC3418140

[B40] Gong K. W. Zhao W. Li N. Barajas B. Kleinman M. Sioutas C. Horvath S. Lusis A. J. Nel A. Araujo J. A. (2007). Air-pollutant chemicals and oxidized lipids exhibit genome-wide synergistic effects on endothelial cells. Genome Biol. 8, R149 10.1186/gb-2007-8-7-r149 17655762 PMC2323217

[B41] Hancock W. W. Buelow R. Sayegh M. H. Turka L. A. (1998). Antibody-induced transplant arteriosclerosis is prevented by graft expression of anti-oxidant and anti-apoptotic genes. Nat. Med. 4, 1392–1396 10.1038/3982 9846576

[B42] Harrison P. M. Arosio P. (1996). The ferritins: molecular properties, iron storage function and cellular regulation. Biochim. Biophys. Acta 1275, 161–203 10.1016/0005-2728(96)00022-9 8695634

[B43] Hartsfield C. L. (2002). Cross talk between carbon monoxide and nitric oxide. Antioxid. Redox Signal. 4, 301–307 10.1089/152308602753666352 12006181

[B44] Hayashi S. Omata Y. Sakamoto H. Higashimoto Y. Hara T. Sagara Y. Noguchi M. (2004). Characterization of rat heme oxygenase-3 gene. Implication of processed pseudogenes derived from heme oxygenase-2 gene. Gene 336, 241–250 10.1016/j.gene.2004.04.002 15246535

[B45] Hopkins P. N. Wu L. L. Hunt S. C. James B. C. Vincent G. M. Williams R. R. (1996). Higher serum bilirubin is associated with decreased risk for early familial coronary artery disease. Arterioscler. Thromb. Vasc. Biol. 16, 250–255 10.1161/01.ATV.16.2.250 8620339

[B46] Huang J. Tabbi-Anneni I. Gunda V. Wang L. (2010). Transcription factor Nrf2 regulates SHP and lipogenic gene expression in hepatic lipid metabolism. Am. J. Physiol. Gastrointest. Liver Physiol. 299, G1211–G1221 10.1152/ajpgi.00322.2010 20930048 PMC3006243

[B47] Ishii T. Itoh K. Ruiz E. Leake D. S. Unoki H. Yamamoto M. Mann G. E. (2004). Role of Nrf2 in the regulation of CD36 and stress protein expression in murine macrophages: activation by oxidatively modified LDL and 4-hydroxynonenal. Circ. Res. 94, 609–616 10.1161/01.RES.0000119171.44657.45 14752028

[B48] Ishikawa K. Navab M. Lusis A. J. (2012). Vascultiis, atherosclerosis, and altered HDL composition in heme-oxygenase-1-knockout mice. Int. J. Hypertens. 10.1155/2012/948203 PMC3296294 22518297

[B49] Ishikawa K. Sugawara D. Goto J. Watanabe Y. Kawamura K. Shiomi M. Itabe H. Maruyama Y. (2001a). Heme oxygenase-1 inhibits atherogenesis in Watanabe heritable hyperlipidemic rabbits. Circulation 104, 1831–1836 10.1161/hc3901.095897 11591622

[B50] Ishikawa K. Sugawara D. Wang X. Suzuki K. Itabe H. Maruyama Y. Lusis A. J. (2001b). Heme oxygenase-1 inhibits atherosclerotic lesion formation in ldl-receptor knockout mice. Circ. Res. 88, 506–512 10.1161/01.RES.88.5.506 11249874

[B51] Jeney V. Balla J. Yachie A. Varga Z. Vercellotti G. M. Eaton J. W. Balla G. (2002). Pro-oxidant and cytotoxic effects of circulating heme. Blood 100, 879–887 10.1182/blood.V100.3.879 12130498

[B52] Juan S. H. Lee T. S. Tseng K. W. Liou J. Y. Shyue S. K. Wu K. K. Chau L. Y. (2001). Adenovirus-mediated heme oxygenase-1 gene transfer inhibits the development of atherosclerosis in apolipoprotein E-deficient mice. Circulation 104, 1519–1525 10.1161/hc3801.095663 11571246

[B53] Juckett M. B. Balla J. Balla G. Jessurun J. Jacob H. S. Vercellotti G. M. (1995). Ferritin protects endothelial cells from oxidized low density lipoprotein in vitro. Am. J. Pathol. 147, 782–789 7677189 PMC1870976

[B54] Kadir F. H. Moore G. R. (1990). Haem binding to horse spleen ferritin. FEBS Lett. 276, 81–84 10.1016/0014-5793(90)80512-H 2265717

[B55] Kadl A. Meher A. K. Sharma P. R. Lee M. Y. Doran A. C. Johnstone S. R. Elliott M. R. Gruber F. Han J. Chen W. Kensler T. Ravichandran K. S. Isakson B. E. Wamhoff B. R. Leitinger N. (2010). Identification of a novel macrophage phenotype that develops in response to atherogenic phospholipids via Nrf2. Circ. Res. 107, 737–746 10.1161/CIRCRESAHA.109.215715 20651288 PMC2941538

[B56] Kaneda H. Ohno M. Taguchi J. Togo M. Hashimoto H. Ogasawara K. Aizawa T. Ishizaka N. Nagai R. (2002). Heme oxygenase-1 gene promoter polymorphism is associated with coronary artery disease in Japanese patients with coronary risk factors. Arterioscler. Thromb. Vasc. Biol. 22, 1680–1685 10.1161/01.ATV.0000033515.96747.6F 12377749

[B57] Kapitulnik J. Maines M. D. (2009). Pleiotropic functions of biliverdin reductase: cellular signaling and generation of cytoprotective and cytotoxic bilirubin. Trends Pharmacol. Sci. 30, 129–137 10.1016/j.tips.2008.12.003 19217170

[B58] Kato H. Amersi F. Buelow R. Melinek J. Coito A. J. Ke B. Busuttil R. W. Kupiec-Weglinski J. W. (2001). Heme oxygenase-1 overexpression protects rat livers from ischemia/reperfusion injury with extended cold preservation. Am. J. Transplant. 1, 121–128 10.1034/j.1600-6143.2001.10205.x 12099359

[B59] Kawashima A. Oda Y. Yachie A. Koizumi S. Nakanishi I. (2002). Heme oxygenase-1 deficiency: the first autopsy case. Hum. Pathol. 33, 125–130 10.1053/hupa.2002.30217 11823983

[B60] Ke B. Shen X. D. Melinek J. Gao F. Ritter T. Volk H. D. Busuttil R. W. Kupiec-Weglinski J. W. (2001). Heme oxygenase-1 gene therapy: a novel immunomodulatory approach in liver allograft recipients? Transplant. Proc. 33, 581–582 10.1016/S0041-1345(00)02151-5 11266967

[B61] Ke B. B. Shen X. D. Melinek J. Busuttil R. W. Kupiec-Weglinski J. W. (2000). Heme oxygenase-1 gene therapy upregulates TH2-dependent expression of protective molecules in liver allograft recipents: implications for novel immunomodulatory approach. Transplantation 69, S280 10.1097/00007890-200004271-00648

[B62] Khoo N. K. Freeman B. A. (2010) Electrophilic nitro-fatty acids: anti-inflammatory mediators in the vascular compartment. Curr. Opin. Pharmacol. 10, 179–184 20080062 10.1016/j.coph.2009.11.003 PMC2843800

[B63] Kim H. P. Wang X. Galbiati F. Ryter S. W. Choi A. M. (2004). Caveolae compartmentalization of heme oxygenase-1 in endothelial cells. FASEB J. 18, 1080–1089 10.1096/fj.03-1391com 15226268

[B64] Krausgruber T. Blazek K. Smallie T. Alzabin S. Lockstone H. Sahgal N. Hussell T. Feldmann M. Udalova I. A. (2011). IRF5 promotes inflammatory macrophage polarization and TH1-TH17 responses. Nat. Immunol. 12, 231–238 10.1038/ni.1990 21240265

[B65] Kruger A. L. Peterson S. Turkseven S. Kaminski P. M. Zhang F. F. Quan S. Wolin M. S. Abraham N. G. (2005). D-4F induces heme oxygenase-1 and extracellular superoxide dismutase, decreases endothelial cell sloughing, and improves vascular reactivity in rat model of diabetes. Circulation 111, 3126–3134 10.1161/CIRCULATIONAHA.104.517102 15939814

[B66] Kruger A. L. Peterson S. J. Schwartzman M. L. Fusco H. Mcclung J. A. Weiss M. Shenouda S. Goodman A. I. Goligorsky M. S. Kappas A. Abraham N. G. (2006). Up-regulation of heme oxygenase provides vascular protection in an animal model of diabetes through its antioxidant and antiapoptotic effects. J. Pharmacol. Exp. Ther. 319, 1144–1152 10.1124/jpet.106.107482 16959961

[B67] Lamb N. J. Quinlan G. J. Mumby S. Evans T. W. Gutteridge J. M. (1999). Haem oxygenase shows pro-oxidant activity in microsomal and cellular systems: implications for the release of low-molecular-mass iron. Biochem. J. 344(Pt 1), 153–158 10.1042/0264-6021:3440153 10548545 PMC1220625

[B68] Laukkanen M. O. Kivela A. Rissanen T. Rutanen J. Karkkainen M. K. Leppanen O. Brasen J. H. Yla-Herttuala S. (2002). Adenovirus-mediated extracellular superoxide dismutase gene therapy reduces neointima formation in balloon-denuded rabbit aorta. Circulation 106, 1999–2003 10.1161/01.CIR.0000031331.05368.9D 12370226

[B69] Lee T. S. Tsai H. L. Chau L. Y. (2003). Induction of heme oxygenase-1 expression in murine macrophages is essential for the anti-inflammatory effect of low dose 15-deoxy-Delta(12,14)-prostaglandin J(2). J. Biol. Chem. 278, 19325–19330 10.1074/jbc.M210063200 12642589

[B70] Lerner-Marmarosh N. Shen J. Torno M. D. Kravets A. Hu Z. Maines M. D. (2005). Human biliverdin reductase: a member of the insulin receptor substrate family with serine/threonine/tyrosine kinase activity. Proc. Natl. Acad. Sci. U.S.A. 102, 7109–7114 10.1073/pnas.0502173102 15870194 PMC1088173

[B71] Levonen A. L. Inkala M. Heikura T. Jauhiainen S. Jyrkkanen H. K. Kansanen E. Maatta K. Romppanen E. Turunen P. Rutanen J. Yla-Herttuala S. (2007). Nrf2 gene transfer induces antioxidant enzymes and suppresses smooth muscle cell growth in vitro and reduces oxidative stress in rabbit aorta in vivo. Arterioscler. Thromb. Vasc. Biol. 27, 741–747 10.1161/01.ATV.0000258868.80079.4d 17255530

[B72] Lewis P. Stefanovic N. Pete J. Calkin A. C. Giunti S. Thallas-Bonke V. Jandeleit-Dahm K. A. Allen T. J. Kola I. Cooper M. E. De Haan J. B. (2007). Lack of the antioxidant enzyme glutathione peroxidase-1 accelerates atherosclerosis in diabetic apolipoprotein E-deficient mice. Circulation 115, 2178–2187 10.1161/CIRCULATIONAHA.106.664250 17420349

[B73] Li T. Tian H. Zhao Y. An F. Zhang L. Zhang J. Peng J. Zhang Y. Guo Y. (2011). Heme oxygenase-1 inhibits progression and destabilization of vulnerable plaques in a rabbit model of atherosclerosis. Eur. J. Pharmacol. 672, 143–152 10.1016/j.ejphar.2011.09.188 22004613

[B74] Lin Q. Weis S. Yang G. Weng Y. H. Helston R. Rish K. Smith A. Bordner J. Polte T. Gaunitz F. Dennery P. A. (2007). Heme oxygenase-1 protein localizes to the nucleus and activates transcription factors important in oxidative stress. J. Biol. Chem. 282, 20621–20633 10.1074/jbc.M700886200 17430897

[B75] Lin Q. S. Weis S. Yang G. Zhuang T. Abate A. Dennery P. A. (2008). Catalytic inactive heme oxygenase-1 protein regulates its own expression in oxidative stress. Free Radic. Biol. Med. 44, 847–855 10.1016/j.freeradbiomed.2007.11.012 18154739 PMC6503848

[B76] Liu D. He Z. Wu L. Fang Y. (2012). Effects of induction/inhibition of endogenous heme oxygenase-1 on lipid metabolism, endothelial function, and atherosclerosis in rabbits on a high fat diet. J. Pharmacol. Sci. 118, 14–24 10.1254/jphs.11181FP 32092834

[B77] Liu X. M. Chapman G. B. Wang H. Durante W. (2002). Adenovirus-mediated heme oxygenase-1 gene expression stimulates apoptosis in vascular smooth muscle cells. Circulation 105, 79–84 10.1161/01.CIR.0000015571.10496.76 11772880

[B78] Llodra J. Angeli V. Liu J. Trogan E. Fisher E. A. Randolph G. J. (2004). Emigration of monocyte-derived cells from atherosclerotic lesions characterizes regressive, but not progressive, plaques. Proc. Natl. Acad. Sci. U.S.A. 101, 11779–11784 10.1073/pnas.0403259101 15280540 PMC511052

[B79] Lusis A. J. (2000). Atherosclerosis. Nature 407, 233–241 10.1038/35025203 11001066 PMC2826222

[B80] Maines M. D. (1997). The heme oxygenase system: a regulator of second messenger gases. Annu. Rev. Pharmacol. Toxicol. 37, 517–554 10.1146/annurev.pharmtox.37.1.517 9131263

[B81] Manthey H. Zernecke A. (2011). Dendritic cells in atherosclerosis: functions in immune regulation and beyond. Thromb. Haemost. 106 10.1160/TH11-05-0296 21901235

[B82] Minamino T. Christou H. Hsieh C. M. Liu Y. X. Dhawan V. Abraham N. G. Perrella M. A. Mitsialis S. A. Kourembanas S. (2001). Targeted expression of heme oxygenase-1 prevents the pulmonary inflammatory and vascular responses to hypoxia. Proc. Natl. Acad. Sci. U.S.A. 98, 8798–8803 10.1073/pnas.161272598 11447290 PMC37515

[B83] Morita T. (2005). Heme oxygenase and atherosclerosis. Arterioscler. Thromb. Vasc. Biol. 25, 1786–1795 10.1161/01.ATV.0000178169.95781.49 16020746

[B84] Morita T. Mitsialis S. A. Koike H. Liu Y. Kourembanas S. (1997). Carbon monoxide controls the proliferation of hypoxic vascular smooth muscle cells. J. Biol. Chem. 272, 32804–32809 10.1074/jbc.272.34.21002 9407056

[B85] Morita T. Perrella M. A. Lee M. E. Kourembanas S. (1995). Smooth muscle cell-derived carbon monoxide is a regulator of vascular cGMP. Proc. Natl. Acad. Sci. U.S.A. 92, 1475–1479 10.1073/pnas.92.5.1475 7878003 PMC42542

[B86] Nagy E. K. Eaton J. W. Jeney V. R. Soares M. P. Varga Z. Galajda Z. N. Szentmiklósi J. Z. Méhes G. B. Csonka T. S. Smith A. Vercellotti G. M. Balla G. R. Balla J. Z. (2010). Red cells, hemoglobin, heme, iron, and atherogenesis. Arterioscler. Thromb. Vasc. Biol. 30, 1347–1353 10.1161/ATVBAHA.110.206433 20378845 PMC2893144

[B87] Neuzil J. Stocker R. (1994). Free and albumin-bound bilirubin are efficient co-antioxidants for alpha-tocopherol, inhibiting plasma and low density lipoprotein lipid peroxidation. J. Biol. Chem. 269, 16712–16719 8206992

[B88] Novotny L. Vitek L. (2003). Inverse relationship between serum bilirubin and atherosclerosis in men: a meta-analysis of published studies. Exp. Biol. Med. 228, 568–571 10.1177/15353702-0322805-29 12709588

[B89] Ollinger R. Bilban M. Erat A. Froio A. Mcdaid J. Tyagi S. Csizmadia E. Graca-Souza A. V. Liloia A. Soares M. P. Otterbein L. E. Usheva A. Yamashita K. Bach F. H. (2005). Bilirubin: a natural inhibitor of vascular smooth muscle cell proliferation. Circulation 112, 1030–1039 10.1161/CIRCULATIONAHA.104.528802 16087796

[B90] Ollinger R. Yamashita K. Bilban M. Erat A. Kogler P. Thomas M. Csizmadia E. Usheva A. Margreiter R. Bach F. H. (2007). Bilirubin and biliverdin treatment of atherosclerotic diseases. Cell Cycle 6, 39–43 10.4161/cc.6.1.3700 17245120

[B91] Orozco L. D. Kapturczak M. H. Barajas B. Wang X. Weinstein M. M. Wong J. Deshane J. Bolisetty S. Shaposhnik Z. Shih D. M. Agarwal A. Lusis A. J. Araujo J. A. (2007). Heme oxygenase-1 expression in macrophages plays a beneficial role in atherosclerosis. Circ. Res. 100, 1703–1711 10.1161/CIRCRESAHA.107.151720 17495224

[B92] Otterbein L. E. Bach F. H. Alam J. Soares M. Tao Lu H. Wysk M. Davis R. J. Flavell R. A. Choi A. M. (2000). Carbon monoxide has anti-inflammatory effects involving the mitogen-activated protein kinase pathway. Nat. Med. 6, 422–428 10.1038/74680 10742149

[B93] Otterbein L. E. Zuckerbraun B. S. Haga M. Liu F. Song R. Usheva A. Stachulak C. Bodyak N. Smith R. N. Csizmadia E. Tyagi S. Akamatsu Y. Flavell R. J. Billiar T. R. Tzeng E. Bach F. H. Choi A. M. Soares M. P. (2003). Carbon monoxide suppresses arteriosclerotic lesions associated with chronic graft rejection and with balloon injury. Nat. Med. 9, 183–190 10.1038/nm817 12539038

[B94] Pamplona A. Ferreira A. Balla J. Jeney V. Balla G. Epiphanio S. Chora A. Rodrigues C. D. Gregoire I. P. Cunha-Rodrigues M. Portugal S. Soares M. P. Mota M. M. (2007). Heme oxygenase-1 and carbon monoxide suppress the pathogenesis of experimental cerebral malaria. Nat. Med. 13, 703–710 10.1038/nm1586 17496899

[B95] Park J. G. Yoo J. Y. Jeong S. J. Choi J. H. Lee M. R. Lee M. N. Hwa Lee J. Kim H. C. Jo H. Yu D. Y. Kang S. W. Rhee S. G. Lee M. H. Oh G. T. (2011). Peroxiredoxin 2 deficiency exacerbates atherosclerosis in apolipoprotein E-deficient mice. Circ. Res. 109, 739–749 10.1161/CIRCRESAHA.111.245530 21835911 PMC5474748

[B96] Phelan D. Winter G. M. Rogers W. J. Lam J. C. Denison M. S. (1998). Activation of the Ah receptor signal transduction pathway by bilirubin and biliverdin. Arch. Biochem. Biophys. 357, 155–163 10.1006/abbi.1998.0814 9721195

[B97] Philippidis P. Mason J. C. Evans B. J. Nadra I. Taylor K. M. Haskard D. O. Landis R. C. (2004). Hemoglobin scavenger receptor CD163 mediates interleukin-10 release and heme oxygenase-1 synthesis - Antiinflammatory monocyte-macrophage responses in vitro, in resolving skin blisters in vivo, and after cardiopulmonary bypass surgery. Circ. Res. 94, 119–126 10.1161/01.RES.0000109414.78907.F9 14656926

[B98] Pittock S. T. Norby S. M. Grande J. P. Croatt A. J. Bren G. D. Badley A. D. Caplice N. M. Griffin M. D. Nath K. A. (2005). MCP-1 is up-regulated in unstressed and stressed HO-1 knockout mice: pathophysiologic correlates. Kidney Int. 68, 611–622 10.1111/j.1523-1755.2005.00439.x 16014038

[B99] Poss K. D. Tonegawa S. (1997). Heme oxygenase 1 is required for mammalian iron reutilization. Proc. Natl. Acad. Sci. U.S.A. 94, 10919–10924 10.1073/pnas.94.20.10919 9380735 PMC23531

[B100] Romanoski C. E. Che N. Yin F. Mai N. Pouldar D. Civelek M. Pan C. Lee S. Vakili L. Yang W. P. Kayne P. Mungrue I. N. Araujo J. A. Berliner J. A. Lusis A. J. (2011). Network for activation of human endothelial cells by oxidized phospholipids. Circ. Res. 109, E27–U52 10.1161/CIRCRESAHA.111.241869 21737788 PMC3163234

[B101] Ryter S. W. Tyrrell R. M. (2000). The heme synthesis and degradation pathways: role in oxidant sensitivity. Heme oxygenase has both pro- and antioxidant properties. Free Radic. Biol. Med. 28, 289–309 10.1016/S0891-5849(99)00223-3 11281297

[B102] Sacerdoti D. Colombrita C. Ghattas M. H. Ismaeil E. F. Scapagnini G. Bolognesi M. Volti G. L. Abraham N. G. (2005). Heme oxygenase-1 transduction in endothelial cells causes downregulation of monocyte chemoattractant protein-1 and of genes involved in inflammation and growth. Cell. Mol. Biol. 51, 363–370 16309586

[B103] Sachais B. S. Turrentine T. Mckenna J. M. D. Rux A. H. Rader D. Kowalska M. A. (2007). Elimination of platelet factor 4 (PF4) from platelets reduces atherosclerosis in C57BI/6 and apoE(-/-) mice. Thromb. Haemost. 98, 1108–1113 18000617

[B104] Sarady J. K. Otterbein S. L. Liu F. Otterbein L. E. Choi A. M. (2002). Carbon monoxide modulates endotoxin-induced production of granulocyte macrophage colony-stimulating factor in macrophages. Am. J. Respir. Cell Mol. Biol. 27, 739–745 12444034 10.1165/rcmb.4816

[B105] Schillinger M. Exner M. Mlekusch W. Domanovits H. Huber K. Mannhalter C. Wagner O. Minar E. (2002). Heme oxygenase-1 gene promoter polymorphism is associated with abdominal aortic aneurysm. Thromb. Res. 106, 131–136 10.1016/S0049-3848(02)00100-7 12182912

[B106] Schwertner H. A. (1998). Association of smoking and low serum bilirubin antioxidant concentrations. Atherosclerosis 136, 383–387 10.1016/S0021-9150(97)00232-3 9543110

[B107] Schwertner H. A. Jackson W. G. Tolan G. (1994). Association of low serum concentration of bilirubin with increased risk of coronary artery disease. Clin. Chem. 40, 18–23 8287538

[B108] Schwertner H. A. Vitek L. (2008). Gilbert syndrome, UGT1A1*28 allele, and cardiovascular disease risk: possible protective effects and therapeutic applications of bilirubin. Atherosclerosis 198, 1–11 10.1016/j.atherosclerosis.2008.01.001 18343383

[B109] Sinal C. J. Bend J. R. (1997). Aryl hydrocarbon receptor-dependent induction of cyp1a1 by bilirubin in mouse hepatoma hepa 1c1c7 cells. Mol. Pharmacol. 52, 590–599 9380021 10.1124/mol.52.4.590

[B110] Singleton J. W. Laster L. (1965). Biliverdin reductase of guinea pig liver. J. Biol. Chem. 240, 4780–4789 4378982

[B111] Siow R. C. Sato H. Mann G. E. (1999). Heme oxygenase-carbon monoxide signalling pathway in atherosclerosis: anti-atherogenic actions of bilirubin and carbon monoxide? Cardiovasc. Res. 41, 385–394 10.1016/S0008-6363(98)00278-8 10341838

[B112] Soares M. P. Bach F. H. (2009). Heme oxygenase-1: from biology to therapeutic potential. Trends. Mol. Med. 15, 50–58 10.1016/j.molmed.2008.12.004 19162549

[B113] Soares M. P. Lin Y. Anrather J. Csizmadia E. Takigami K. Sato K. Grey S.T. Colvin R. B. Choi A. M. Poss K. D. Bach F. H. (1998). Expression of heme oxygenase-1 can determine cardiac xenograft survival. Nat. Med. 4, 1073–1077 10.1038/2063 9734404

[B114] Sriram V. Patterson C. (2001). Cell cycle in vasculoproliferative diseases: potential interventions and routes of delivery. Circulation 103, 2414–2419 10.1161/01.CIR.103.19.2414 11352893

[B115] Stocker R. Keaney J. F. Jr. (2004). Role of oxidative modifications in atherosclerosis. Physiol. Rev. 84, 1381–1478 10.1152/physrev.00047.2003 15383655

[B116] Stocker R. Yamamoto Y. Mcdonagh A. F. Glazer A. N. Ames B. N. (1987). Bilirubin is an antioxidant of possible physiological importance. Science 235, 1043–1046 10.1126/science.3029864 3029864

[B117] Sussan T. E. Jun J. Thimmulappa R. Bedja D. Antero M. Gabrielson K. L. Polotsky V. Y. Biswal S. (2008). Disruption of Nrf2, a key inducer of antioxidant defenses, attenuates ApoE-mediated atherosclerosis in mice. PLoS One 3, e3791 10.1371/journal.pone.0003791 19023427 PMC2582492

[B118] Taha H. Skrzypek K. Guevara I. Nigisch A. Mustafa S. Grochot-Przeczek A. Ferdek P. Was H. Kotlinowski J. Kozakowska M. Balcerczyk A. Muchova L. Vitek L. Weigel G. Dulak J. Jozkowicz A. (2010). Role of heme oxygenase-1 in human endothelial cells: lesson from the promoter allelic variants. Arterioscler. Thromb. Vasc. Biol. 30, 1634–1641 10.1161/ATVBAHA.110.207316 20508205 PMC2906705

[B119] Tsuchihashi S. Livhits M. Zhai Y. Busuttil R. W. Araujo J. A. Kupiec-Weglinski J. W. (2006). Basal rather than induced heme oxygenase-1 levels are crucial in the antioxidant cytoprotection. J. Immunol. 177, 4749–4757 16982915 10.4049/jimmunol.177.7.4749

[B120] Tulis D. A. Durante W. Liu X. Evans A. J. Peyton K. J. Schafer A. I. (2001). Adenovirus-mediated heme oxygenase-1 gene delivery inhibits injury-induced vascular neointima formation. Circulation 104, 2710–2715 10.1161/hc4701.099585 11723024

[B121] Wang L. J. Lee T. S. Lee F. Y. Pai R. C. Chau L. Y. (1998). Expression of heme oxygenase-1 in atherosclerotic lesions. Am. J. Pathol. 152, 711–720 9502413 PMC1858397

[B122] Wang X. M. Kim H. P. Nakahira K. Ryter S. W. Choi A. M. (2009). The heme oxygenase-1/carbon monoxide pathway suppresses TLR4 signaling by regulating the interaction of TLR4 with caveolin-1. J. Immunol. 182, 3809–3818 10.4049/jimmunol.0803738 19265160

[B123] Weis N. Weigert A. Von Knethen A. Brune B. (2009). Heme oxygenase-1 contributes to an alternative macrophage activation profile induced by apoptotic cell supernatants. Mol. Biol. Cell 20, 1280–1288 10.1091/mbc.E08-10-1005 19129475 PMC2649271

[B124] Wilson H. M. (2010). Macrophages heterogeneity in atherosclerosis - implications for therapy. J. Cell. Mol. Med. 14, 2055–2065 10.1111/j.1582-4934.2010.01121.x 20629993 PMC3822996

[B125] Wu D. Nishimura N. Kuo V. Fiehn O. Shahbaz S. Van Winkle L. Matsumura F. Vogel C. F. (2011). Activation of aryl hydrocarbon receptor induces vascular inflammation and promotes atherosclerosis in apolipoprotein E-/- mice. Arterioscler. Thromb. Vasc. Biol. 31, 1260–1267 10.1161/ATVBAHA.110.220202 21441140 PMC3098318

[B126] Wu T. W. Fung K. P. Wu J. Yang C. C. Weisel R. D. (1996). Antioxidation of human low density lipoprotein by unconjugated and conjugated bilirubins. Biochem. Pharmacol. 51, 859–862 10.1016/0006-2952(95)02182-5 8602883

[B127] Wuttge D. M. Eriksson P. Sirsjö A. Hansson G. K. Stemme S. (2001). Expression of interleukin-15 in mouse and human atherosclerotic lesions. Am. J. Pathol. 159, 417 10.1016/S0002-9440(10)61712-9 11485899 PMC1850554

[B128] Yachie A. Niida Y. Wada T. Igarashi N. Kaneda H. Toma T. Ohta K. Kasahara Y. Koizumi S. (1999). Oxidative stress causes enhanced endothelial cell injury in human heme oxygenase-1 deficiency. J. Clin. Invest. 103, 129–135 10.1172/JCI4165 9884342 PMC407858

[B129] Yamada N. Yamaya M. Okinaga S. Nakayama K. Sekizawa K. Shibahara S. Sasaki H. (2000). Microsatellite polymorphism in the heme oxygenase-1 gene promoter is associated with susceptibility to emphysema. Am. J. Hum. Genet. 66, 187–195 10.1086/302729 10631150 PMC1288325

[B130] Yamashita K. Mcdaid J. Ollinger R. Tsui T. Y. Berberat P. O. Usheva A. Csizmadia E. Smith R. N. Soares M. P. Bach F. H. (2004). Biliverdin, a natural product of heme catabolism, induces tolerance to cardiac allografts. FASEB J. 18, 765–767 14977878 10.1096/fj.03-0839fje

[B131] Yan Z. Q. Hansson G. K. (2007). Innate immunity, macrophage activation, and atherosclerosis. Immunol. Rev. 219, 187–203 10.1111/j.1600-065X.2007.00554.x 17850490

[B132] Yet S. F. Layne M. D. Liu X. Chen Y. H. Ith B. Sibinga N. E. Perrella M. A. (2003). Absence of heme oxygenase-1 exacerbates atherosclerotic lesion formation and vascular remodeling. FASEB J. 17, 1759–1761 12958201 10.1096/fj.03-0187fje

[B133] Yilmaz A. Lochno M. Traeg F. Cicha I. Reiss C. Stumpf C. Raaz D. Anger T. Amann K. Probst T. Ludwig J. Daniel W. G. Garlichs C. D. (2004). Emergence of dendritic cells in rupture-prone regions of vulnerable carotid plaques. Atherosclerosis 176, 101–110 10.1016/j.atherosclerosis.2004.04.027 15306181

